# Systemic Modulators: Potential Mechanism for the 5-HT System to Mediate Exercise Amelioration in Alzheimer's Disease

**DOI:** 10.14336/AD.2024.0834

**Published:** 2025-09-30

**Authors:** Qingxu Wu, Qiang He, Xianliang Zhang, Si Chen, Xiangli Xue

**Affiliations:** ^1^School of Physical Education, Shandong University, Jinan, Shandong, China.; ^2^School of Nursing and Rehabilitation, Cheeloo College of Medicine, Shandong University, Jinan, Shandong, China.

**Keywords:** 5-HT, Alzheimer’s disease;, exercise, cognitive function, central, peripheral

## Abstract

As a neurodegenerative disease closely related to age-related changes, Alzheimer's disease (AD) is rapidly becoming one of the most resource-intensive and deadly diseases of this century. As a systemic neurotransmitter system with widespread distribution throughout the central and peripheral nervous systems, the 5-hydroxytryptamine (5-HT) system not only plays an important role in antidepressant therapy but also shows potential value in improving AD symptoms. The 5-HT system may facilitate the prevention and treatment of AD by impacting its pathological processes through various pathways, such as the regulation of Aβ deposition, hyperphosphorylation of Tau, central and peripheral neuroinflammation, and the interactions with the cholinergic and BDNF systems. In addition, regular exercise, as a non-pharmacological intervention, provides systemic and multi-level physical health benefits. Given the high sensitivity of the 5-HT system to exercise, this paper reviews its crucial role and potential mechanisms in alleviating AD through exercise. From perspective of the integrative biology of exercise, we propose several crosstalk mechanisms between the peripheral and central systems mediated by the 5-HT system. These mechanisms serve as a bridge for the treatment of AD and offer novel ideas and strategies for future therapeutic approaches.

## 1. Introduction

Alzheimer's disease (AD), a leading cause of dementia, is increasingly recognized as one of the most resource-intensive and lethal diseases of this century as the global population ages. Its main pathological features include amyloid beta (Aβ) protein deposition, abnormal aggregation of Tau protein leading to neurofibrillary tangles, neuroinflammation, cholinergic dysfunction, and synaptic loss. These features lead to clinical symptoms such as declining learning, memory, and cognition [1, 2]. Despite extensive research into treatments for AD, no pharmacological intervention or therapeutic regimen has yet achieved complete reversal or effectively halted disease progression. Importantly, depression frequently coexists with AD, and the two conditions share several pathological features. Furthermore, evidence indicates a causal relationship between depression and AD [3, 4], which highlights the potential benefits of effective depression treatments in the management of AD. 5-hydroxytryptamine (5-HT), commonly referred to as serotonin, is a crucial neurotransmitter involved in the mechanisms of antidepressants. Post-mortem analysis of AD patient brain tissue has revealed reduced levels of 5-HT and its metabolites, alongside dysfunction of the entire 5-HT system [5, 6]. These findings highlight a significant link between 5-HT and AD, suggesting a new avenue for exploring the mechanisms of AD treatment.

The 5-HT system primarily comprises 5-HT, 5-HT neurons, 5-HT receptors (5-HTRs), 5-HT transporter (SERT), and pathways for 5-HT synthesis and metabolism. 5-HT is present not only in the central and peripheral nervous systems but also in non-neuronal systems including the gut, lymphatic system, and blood [7-9]. Its effects on behavior are mediated through a family of specific 5-HTRs. Seven subtypes have been identified, with only the 5-HT_3_R linked to ligand-gated ion channels; the other six subtypes are coupled to G proteins [10]. Initially, drugs targeting 5-HT, such as 5-HTR agonists, antagonists, and selective 5-HT reuptake inhibitors (SSRIs), were primarily used to treat depression. Recent studies have indicated that disruptions in the 5-HT system are closely associated with memory loss in AD [11]. Additionally, this class of drugs has been shown to play an active role in the treatment of AD [12, 13].

In recent years, exercise has shown significant positive effects as a non-pharmacological treatment for AD; however, the specific mechanisms remain incompletely understood [14]. Existing studies have shown that exercise significantly regulates the 5-HT system, which plays a pivotal role in AD-related phenomena such as exercise-induced neurogenesis and memory enhancement [15-17]. The integrative biology of exercise posits that it disrupts the body's initial homeostasis of cells, tissues, and organs, thereby establishing a new dynamic equilibrium. This disruption and reshaping of systemic homeostasis not only benefit skeletal muscle but also yield positive effects on various organs and systems throughout the body [18].

Based on the positive impact demonstrated by the 5-HT system in improving AD through exercise and its sensitivity to exercise, this review focuses on the critical role of the 5-HT system in the treatment of AD and explores the mediating role in the effects of exercise on AD. These insights provide a new theoretical foundation for the prevention and treatment of AD and other cognitive disorders.

## 2. The pathological mechanisms through which the 5-HT system ameliorates AD

### 2.1 5-HT system regulates Aβ production and clearance

The Aβ hypothesis proposes that the abnormal aggregation of Aβ in brain tissue initiates Tau tangle formation, neuronal dysfunction, and cognitive decline, marking the onset of AD [19]. Substantial evidence indicates that 5-HT regulates both the production and clearance of Aβ through diverse pathways, involving a broad spectrum of specific 5-HTRs, with notable roles played by 5-HT_2_R, 5-HT_4_R, and 5-HT_6_R.

#### 2.1.1 5-HTRs

Firstly, the activation of the 5-HT_2A_R with TCB-2 (5 μg/μl, icv) has been shown to enhance cognitive function by reducing Aβ deposition in the hippocampus and cortex of AD mice. However, the precise mechanism of action remains unclear [20]. Secondly, the antagonism of 5-HT_2A_R by desloratadine has been demonstrated to enhance cellular autophagy and microglial phagocytosis. This effect is achieved through the activation of the cyclic adenosine monophosphate (cAMP)/cAMP-dependent protein kinase A (PKA) pathway, along with its downstream cAMP-response element binding protein (CREB)/sirtuin 1 (Sirt1) and CREB/glucocorticoid receptor (GR)/Toll-like receptors 2 and 4 (TLR2/4) pathways. This mechanism leads to a reduction in Aβ levels in the hippocampal CA1 region of APP/PS1 mice, ultimately improving spatial learning and memory capacity mediated by the hippocampus and prefrontal cortex (PFC) [21]. Furthermore, the 5-HT_2A_R inverse agonist Pimavanserin has been demonstrated to reduce Aβ production and deposition in the interstitial fluid (ISF) of AD mice by increasing α-secretase-dependent cleavage of amyloid precursor protein (APP), thereby improving cognitive function and memory performance. This effect is mediated through the N-methyl-D-aspartate receptors (NMDA-Rs)/extracellular signal-regulated kinase (ERK) pathway downstream of 5-HT_2A_R [22].

The 5-HT_4_R plays a crucial role in improving Aβ pathology mechanisms. SSP-002392, an orally administered 5-HT_4_R agonist at a dose of 5 mg/kg, has been shown to enhance the degradation of Aβ by increasing the presence of microglial cells and astrocytes surrounding Aβ deposits in the hippocampus of AD mouse models [23]. Additionally, the 5-HT_4_R influences various cleavage pathways of APP, including α-, β-, and γ-secretase pathways. Activation of the 5-HT_4_R, whether in vivo or in vitro, leads to APP cleavage by α-secretase, releasing neuroprotective soluble amyloid precursor protein alpha (sAPPα). This process indirectly reduces Aβ production and correlates with improved cognitive function [13, 23-29]. The specificity of this effect is evident since it can be inhibited by 5-HT_4_R antagonists [24-27]. The mechanisms through which 5-HT_4_R mediates sAPPα release are diverse, with one such mechanism involving the classical cAMP/PKA signaling pathway. Reverse microdialysis of 5-HT (2 mM) or the 5-HT_4_R agonist ML10302 (400 nM) induces cAMP production in the brains of AD mouse models, subsequently activating PKA and the downstream mitogen-activated protein-ERK kinase (MEK)/ERK pathway. This cascade enhances α-secretase activity, promotes sAPPα release, and reduces Aβ levels in the ISF [13]. Interestingly, the PKA inhibitor H89 did not reduce the increase in sAPPα levels induced by 5-HT via 5-HT4(e)R in cellular experiments [25]. This suggests that the 5-HT-induced increase in sAPPα may not solely depend on PKA, which might exert a negative regulatory influence. Other studies have demonstrated this mechanism, whereby 5-HT regulates the α-secretase pathway of APP through a mechanism that is independent of PKA. 5-HT stimulation of 5-HT_4_R can directly activate the cAMP/exchange protein directly activated by cAMP (Epac)/Ras-related protein (Rap) pathway and downstream Rac, thereby facilitating α-secretase cleavage of APP and promoting the release of sAPPα. This mechanism specifically involves the 5-HT_4(e)_R [26, 27]. Additionally, overexpression of 5-HT_4_R increases sAPPα levels via matrix metalloproteinase-9 (MMP-9) [28]. In addition to regulating the α-secretase pathway, after 37 days of treatment with SSP-002392 (5 mg/kg, p.o.), Aβ production was reduced due to decreased levels of hippocampal BACE1 in hAPP/PS1 mice [23]; similar effects were observed after just 90 minutes of treatment. Additionally, different subtypes of 5-HT_4_R may modulate the β- or γ-secretase pathway of APP [29], necessitating further experimental validation. These studies suggest that 5-HT_4_R may influence APP cleavage via multiple pathways, thereby regulating both Aβ levels and cognitive functions.

Activation or antagonism of 5-HT_6_R shows similar effectiveness in improving Aβ pathology. In PC-12 cells induced by Aβ neurotoxicity, both 5-HT_6_R agonists and antagonists show neuroprotective effects [30]. Cellular experiments demonstrate that SB258585, a selective 5-HT_6_R antagonist, affects multiple targets mediated by 5-HT_6_R, such as β-arrestin2 and cyclin-dependent kinase 5 (CDK5), resulting in a dose-dependent reduction in Aβ production [31]. Animal experiments have revealed an additional mechanism: SB271046, a 5-HT_6_R antagonist (10 mg/kg, i.p.), reduced Aβ_1-42_ levels in the brains of AD mice and improved learning and memory deficits by inhibiting γ-secretase activity via APBA1/2 modulation [32]. Furthermore, overexpression of 5-HT_6_R through treatment with 5-HT reduced Aβ levels in the ISF of AD mice [13].

Although few studies have investigated other 5-HTRs and Aβ, these limited findings suggest their role in modulating Aβ levels. Specifically, treatment with the 5-HT_1A_R antagonist NAD-99 (5 μg/μl, icv) and the 5-HT_3A_R antagonist Tropisetron (0.5 mg/kg, i.p.) reduced Aβ deposition in the hippocampus and cortex of AD mice, thereby improving cognitive function [20, 33]. Additionally, the 5-HT_7_R agonist AS19 (1 μg/μl, icv) decreased Aβ deposition in the brains of AD mice and significantly ameliorated recognition and passive avoidance memory deficits caused by Aβ injection [34].

#### 2.1.2 SSRIs and high tryptophan diet

SSRIs such as fluoxetine, citalopram, escitalopram, and sertraline exert their antidepressant effects by inhibiting SERT, which increases the concentration of 5-HT in the synaptic cleft. These drugs have also shown significant positive effects in the treatment of AD. Clinical, cellular, and animal studies have shown the beneficial effects of SSRIs in reducing Aβ pathology. A retrospective analysis spanning five years revealed significantly lower Aβ deposition in the brains of participants treated with SSRIs compared to those who were not [12]. In cellular studies, fluoxetine, citalopram, and sertraline effectively reduced Aβ production in SK-N-SH cells [31].

Animal studies further confirmed these findings and explored the mechanisms by which SSRIs regulate Aβ. Research indicated that SSRIs could regulate the cleavage of APP through multiple pathways, thereby effectively reducing Aβ levels. Firstly, fluoxetine (5 mg/kg, p.o.) was found to reduce Aβ production by inhibiting APP phosphorylation at the T668 site [35]. Secondly, SSRIs also modulate Aβ levels by increasing α-secretase activity. When researchers administered escitalopram (5 mg/kg) orally after eight hours or citalopram (8 mg/kg/d) for four months to APP/PS1 mice, they observed an increase in α-secretase activity across various brain regions, which reduced Aβ deposition [12, 36]. This effect likely involves the activation of several signaling pathways, including the phosphatidylinositol 3-kinase (PI3K)/protein kinase B (Akt)/glycogen synthase kinase-3β (GSK-3β), Raf-1/MEK/ERK, and c-Jun N-terminal kinase (JNK)/c-Jun pathways [37]. Similar outcomes were observed with a four-month fluoxetine treatment (10 mg/kg/d, i.p.), albeit through a different pathway. This process simultaneously enhances the activity of protein phosphatases 2A (PP2A) and activates Wnt/β-catenin signaling. As a result, α-secretase activity increases, while γ-secretase activity and BACE1 levels are reduced. This ultimately lowers Aβ levels in the hippocampus of AD mice, which subsequently leads to improved spatial learning and memory performance [38]. Additionally, SSRIs can also modulate Aβ levels via other pathways. Fluoxetine (1 μM) reduced high levels of Aβ_40_ and Aβ_42_ in cultured astrocytes, an effect that was counteracted by 5-HT_2_R antagonists. This suggests that fluoxetine's inhibition of Aβ production is likely mediated through the 5-HT_2_R pathway in astrocytes [39]. In a Caenorhabditis elegans (C. elegans) model of AD, administration of fluoxetine at multiple doses reduced Aβ oligomers and alleviated Aβ-induced neurotoxicity via the FOXO transcription factor (DAF-16)-related pathway [40]. Additionally, PKA antagonists blocked the effect of citalopram (10 mg/kg, i.p.) on reducing Aβ levels in the ISF of AD mice, suggesting that citalopram can modulate Aβ levels via PKA-related signaling pathways [13]. Besides SSRIs, a high tryptophan (Trp) diet (hereinafter referred to as HTrp) elevates in vivo 5-HT concentration similarly to SSRIs. HTrp also reduces intraneuronal Aβ load in neurons in the CA1 region of the hippocampus in 3×TgAD mice [41]. In conclusion, SSRIs have demonstrated significant effects on Aβ levels in clinical, cellular, and animal studies, positioning them as a potential strategy for AD treatment. They achieve this by reducing Aβ levels and enhancing cognitive function through the modulation of the APP cleavage process and other signaling pathways. However, in some studies, SSRIs did not show modulation of Aβ [42], which may depend on factors such as route of administration and mode of administration (acute or chronic), as well as the timing of measurement relative to the initiation of drug therapy.

The above studies have robustly shown that the 5-HT system actively enhances AD treatment by modulating Aβ formation, clearance, and homeostasis through diverse pathways.

### 2.2 5-HT system improves Tau pathology

Abnormal Tau modifications, such as hyperphosphorylation, lead to degenerative neuronal lesions or even cell death in the brains of AD patients. These abnormal modifications ultimately result in dementia symptoms, including memory loss and cognitive decline. The 5-HT system actively contributes to alleviating Tau pathology in AD, with GSK-3β playing a pivotal role in this process. Numerous studies have shown that 5-HT can regulate Tau protein phosphorylation levels through the GSK-3β-related pathway and other mechanisms, thereby influencing Tau pathology and cognitive dysfunction. The specific pathways are as follows.

PI3K/Akt/GSK-3β signaling pathway: Li et al. first revealed that the selective 5-HT_1A_R agonist 8-OH-DPAT or fluoxetine increased the levels of phosphorylated GSK-3β Ser9 in the hippocampus, striatum and PFC of mice [43]. Subsequent cellular and animal experiments further validated this pathway and observed reduced levels of Tau phosphorylation and amelioration of cognitive deficits [37, 44-48]. Two mechanisms mediate this process: (i) SSRIs lead to elevated levels of 5-HT in the brain, which in turn activates the 5-HT_1A_R/PI3K/Akt/GSK-3β pathway. (ii) 5-HT can activate the Akt/GSK-3β pathway through a non-5-HT_1A_R-dependent mechanism.

Mammalian target of rapamycin (mTOR)/GSK-3β signaling pathway: In cellular and animal studies, overexpression of 5-HT_6_R has been found to contribute to Tau pathology. This occurs through the activation of mTOR signaling via the classical PI3K/Akt/Ras homolog enriched in brain (Rheb) pathway or occurs through the physical interactions between 5-HT_6_R and mTOR, which in turn enhances GSK-3β activity [48-50].

CDK5/GSK-3β signaling pathway: 5-HT_6_R activates CDK5 signaling independently of agonists [51]. This increased CDK5 phosphorylation then leads to subsequent phosphorylation of GSK-3β, which further enhances total Tau phosphorylation [52]. Similarly, CDK5 is implicated in Tau phosphorylation triggered by 5-HT_7_R activation in mice, which correlates with impaired memory function. Interestingly, GSK-3β does not influence this process [53].

Fyn/GSK-3β signaling pathway: The physical interaction between 5-HT_6_R and Fyn modulates the coupling of 5-HT_6_R to G proteins. This indicates that Fyn may regulate the activation of the 5-HT_6_R pathway by influencing receptor binding to G proteins. Moreover, stimulation of 5-HT_6_R increases Fyn phosphorylation, and higher Fyn expression enhances 5-HT_6_R activity [54]. Collectively, these effects activate the Fyn/GSK-3β pathway [55], which may result in elevated levels of Tau phosphorylation [56].

Other signaling pathway: Increased activity of both ERK and microtubule affinity-regulating kinase 4 (MARK4) induces the deposition and phosphorylation of Tau proteins, which in turn leads to Tau pathology [57]. Overexpression of 5-HT_6_R increases the level of Tau phosphorylation by activating ERK1/2 via a Fyn-dependent pathway and partially through a PKA-dependent pathway [54]. 5-HT is able to ameliorate Tau hyperphosphorylation by inhibiting MARK4 activity [58].

In conclusion, the 5-HT system alleviates Tau pathology and cognitive deficits via the classical PI3K/Akt/GSK-3β pathway. Moreover, 5-HT regulates Tau phosphorylation through various signaling pathways, such as the mTOR/GSK-3β, CDK5/GSK-3β, Fyn/GSK-3β, Fyn-PKA/ERK1/2, and MARK4 pathways, offering crucial insights into potential therapeutic targets for AD. The above studies suggest that inhibiting 5-HT_6_R and 5-HT_7_R could benefit AD treatment.

### 2.3 5-HT system regulates the inflammatory response

Neuroinflammation is a hallmark of AD [59]. It involves the activation of immune cells and the release of inflammatory mediators, which can further activate microglia and astrocytes in the central nervous system (CNS) [60, 61]. This activation leads to increased production of reactive oxygen species (ROS), which can cause oxidative stress and ultimately result in neuronal damage [62]. Additionally, due to the presence of the blood-brain barrier (BBB), peripheral pro-inflammatory cytokines can influence brain tissue via neural, cellular, and humoral pathways [63-65]. Thus, reducing pro-inflammatory factors and enhancing anti-inflammatory factors, both centrally and peripherally, are crucial for mitigating the effects of AD.

#### 2.3.1 Modulation of central inflammation by the 5-HT system

5-HT production and metabolic pathways are closely linked to inflammatory responses. A study found higher serum Kyn/5-HT ratios in healthy individuals, individuals with mild cognitive impairment (MCI), and AD patients. These ratios correlate with elevated levels of peripheral immune markers, such as IL-1ra, IL-12p40, and IL-18. Additionally, these ratios are associated with cognitive decline and grey matter atrophy in areas sensitive to AD [66]. Neuroinflammation and chronic stress increase the expression of tryptophan 2,3-dioxygenase (TDO) and indoleamine 2,3-dioxygenase (IDO). These enzymes activate the kynurenine (Kyn) metabolic pathway of Trp, diverting Trp away from 5-HT synthesis [67]. As a result, there is excessive accumulation of Kyn metabolites in the brain. This accumulation can induce CNS inflammation, synaptic dysfunction, and affect neuronal regeneration and degeneration [68]. Consequently, this process forms a vicious cycle that accelerates the pathological progression of AD. Moreover, 5-HT metabolites, such as melatonin and N-acetylserotonin, enhance mitochondrial oxidative phosphorylation and exhibit anti-inflammatory and antioxidant properties [69]. These findings suggest that neuroinflammation is associated with reduced Trp and 5-HT levels and increased Kyn levels. Mitigating these changes could potentially attenuate neuroinflammation and improve outcomes in AD.

SERT and various 5-HTRs play crucial roles in regulating CNS inflammatory responses. In a transgenic mouse model of familial AD, reduced SERT activity has been linked to CNS inflammation [70]. Additionally, various 5-HTRs modulate CNS inflammation through distinct pathways. Both 5-HT_1_R subtypes, 5-HT_1A_R and 5-HT_1B_R, are significantly associated with Aβ-induced neuroinflammation, as demonstrated in cellular and animal studies. The 5-HT_1A_R antagonist WAY100635 (0.5 mg/kg, i.p.) induces a reduction in the number of microglia, astrocytes, and Iba1^+^ and TNF-α^+^ cells in the hippocampal dentate gyrus of AD mice. This reduction is associated with the activation of nuclear factor κB (NF-κB) and related crosstalk mechanisms. The treatment also leads to a decrease in the expression of inflammation-related proteins such as IKK-β, NF-κB, and p-NF-κB, as well as a decrease in the ratio of p-NF-κB to NF-κB. Ultimately, this results in significant alleviation of neuroinflammation, deficits in short-term memory, and impairments in spatial learning abilities in mice treated with Aβ_1-42_. Furthermore, WAY100635 increased the expression of the inflammation-related protein Tβ4 and elevated IKB-α levels, a crucial regulator of NF-κB, thereby enhancing neuroprotection [71]. In addition to antagonists, the 5-HT_1B_R selective agonist emodin-8-O-β-D-glucopyranoside (EG) suppressed Aβ-induced inflammation in PC12 cells and the SH-SY5Y cell line, reducing cytotoxicity and cell death. In C. elegans model, EG (200 μM) specifically reduced the expression of Aβ-induced immune response genes, such as nhr-57, pqn-5, and col-41. This effect does not indiscriminately inhibit all immune pathways but rather provides targeted protection against AD in the worm [72].

Antagonism of both 5-HT_2A_R and 5-HT_3A_R has demonstrated anti-inflammatory effects in AD mice and is associated with improvements in cognitive deficits. The 5-HT_2A_R antagonist desloratadine (20 mg/kg, i.p.) reduces inflammatory responses in the brains of APP/PS1 mice by activating the cAMP/PKA/CREB/Sirt1 pathway. First, desloratadine decreases the expression of pro-inflammatory cytokines TNF-α and IL-6 and reduces the activation of NLRP3 inflammasomes in the hippocampal CA1 region. Second, it enhances the levels of anti-inflammatory cytokines IL-4 and IL-10. Furthermore, desloratadine effectively inhibits the nuclear translocation of NF-κB in hippocampal microglia and promotes the shift of microglial polarization from the pro-inflammatory M1 phenotype to the anti-inflammatory M2 phenotype. This shift enhances microglial autophagy and facilitates the clearance of Aβ, thus collectively improving cognitive deficits in AD mice [21]. Similarly, the 5-HT_3A_R antagonist tropisetron (0.5 mg/kg, i.p.) reduces glial cell activation and the levels of pro-inflammatory cytokines IL-1β and TNF-α. It also decreases iNOS-positive areas in the cortex and hippocampus of Tg AD mice. This reduction in neuroinflammation accompanies the restoration of presynaptic synaptic vesicle proteins in the hippocampus, which had been lost due to AD pathology, thereby improving cognitive deficits [33].

5-HT_4_R and 5-HT_6_R exhibit positive effects in improving inflammation and cognitive deficits. Studies have demonstrated that administering the 5-HT_4_R agonist RS 67333 (1 mg/kg, i.p.) to 5xFAD mice for three to four months significantly reduces Aβ levels. This treatment results in a significant decrease in hippocampal astrogliosis and microgliosis, likely due to the expression of 5-HT_4_R on hippocampal astrocytes affecting their morphological changes [73]. Additionally, it lowered the levels of inflammatory mediators IL-1β and MCP-1 in the entorhinal cortex, ultimately leading to reduced brain tissue inflammation and reversal of cognitive deficits in the mice. Interestingly, a two-month treatment with RS 67333 (1 mg/kg, i.p.) did not yield these benefits [74, 75]. This suggests that the positive effects—including reduced inflammation and cognitive enhancement—are associated with prolonged activation of 5-HT_4_R. In addition, antagonizing 5-HT_6_R using SB271046 (10 mg/kg, i.p.) similarly improves neuroinflammation and cognitive function in AD mice. However, the specific mechanisms of action remains unclear [32].

In addition, SSRIs can exert significant anti-inflammatory effects in the CNS through different mechanisms. Research has shown that the absence of the anti-inflammatory cytokine TGF-β1 induces neuroinflammation and cognitive deficits associated with AD [76]. Researchers found that fluoxetine (10 mg/kg, i.p.) effectively restores Aβ-induced declines in hippocampal TGF-β1 levels and improves cognitive deficits in AD mice [77]. Escitalopram may mitigate various AD pathologies, including neuroinflammation, by modulating pathways such as PI3K/Akt/GSK-3β, Raf-1/MEK/ERK, and JNK/c-Jun [37]. Additionally, alongside the conventional SSRIs mentioned earlier, traditional Chinese medicine Kai-Xin-san (10 g/kg, p.o.) has demonstrated its ability to reduce neuroinflammation. It achieves this by increasing plasma levels of the neurotransmitter 5-HT and enhancing the activation of the PI3K/Akt/GSK-3β pathway. This action significantly lowers ROS levels and reduces the concentrations of inflammatory factors such as IL-1β and TNF-α in the hippocampus of AD model mice. Consequently, it attenuates neuroinflammation and ROS-induced neuronal apoptosis, ultimately improving learning and memory performance [47] (Table 1).

**Table 1:** Potential mechanisms by which 5-HTRs influence AD-related pathology.

5-HTR	Subtype	Agent	Subject	Program	Potential pathway	AD-related pathological changes	Ref.
**5-HT_1_R**	5-HT_1A_R	8-OH-DPAT (A)	Cell	80 μM, 24 hours	↓: GSK-3β ↑: Akt and PI3K	↓: Tau	[45]
		NAD-99 (AN)	Rat	5 μg/μl, icv, 30 days	NR	↓: Aβ and neuronal apoptosis ↑: BDNF and cognition	[20]
		WAY100635 (AN)	Mice	0.5 mg/kg/d, i.p., 14 days	↑: NF-κB	↓: Neuroinflammation ↑: Cognition	[71]
	5-HT_1B_R	Emodin-8-O-β-D-glucopyranoside (A)	Cell and C. elegans	**Cell**: 0.1 μM, pre-treated 18 hours **C. elegans:** 100, 200 and 500 μM, 56 hours	NR	↓: Aβ neurotoxicity, neuroinflammation and neuronal apoptosis	[72]
**5-HT_2_R**	5-HT_2A_R	TCB-2 (A)	Rat	5 μg/μl, icv, 30 days	NR	↓: Aβ and neuronal loss ↑: BDNF and cognition	[20]
		Desloratadine (AN)	Mice	20 mg/kg/d, i.p., 3.5 months	↑: cAMP, PKA, CREB, GR and TLR2/4	↓: Aβ and neuroinflammation ↑: Synaptic plasticity and integrity	[21]
		Pimavanserin (IN)	Mice	3 mg/kg/d, scip, 4 months	**Relate**: NMDA-Rs and ERK	↓: Aβ ↑: α-secretase	[22]
**5-HT_3_R**	5-HT_3A_R	Tropisetron (AN)	Mice	0.5 mg/kg/d, i.p., 8 weeks	↓: CaN and NFAT	↓: Aβ and neuroinflammation ↑: Cognition	[33]
**5-HT_4_R**		SSP-002392 (A) and RS 67333 (A)	Cell and mice	**SSP-002392** **Cell:** 0.0001-1 μM, 24 hours **Mice:** 5 mg/kg, p.o., 37 days RS 67333 Mice: 2 mg/kg/2 d, p.o., 4 months	NR	↓: Aβ, ADAM17, β-secretase, nicastrin and neuroinflammation ↑: cAMP and cognition	[23]
		RS 67333 (A)	Cell and mice	**Cell**: 0.001-100 μM, 30 min* Mice: 1 mg/kg, i.p., twice a week for 2 or 3 months	NR	↓: Aβ and neuroinflammation ↑: sAPPα and cognition	[74]
			Cell and mice	**Cell**: 3 μM, 8, 24 and 48 hours Mice: 3 mg/kg, i.p., 10 days	↑: MMP-9	↓: Aβ ↑: sAPPα and CTF-α	[28]
			Mice	1 mg/kg, i.p., twice a week for 2 or 4 months	NR	↓: Aβ and neuroinflammation ↑: Cognition	[75]
		ML10302 (A)	Mice	400 nM, rev md, 24 hours	**Relate**: PKA, MEK and ERK	↓: Aβ	[13]
		Prucalopride (A)	Cell	1 μM, 2 hours	↑: cAMP and Epac	↑: sAPPα	[26]
		Prucalopride (A) and ML10302 (A)	Cell	Prucalopride 1 μM, overnight ML10302 1 μM, overnight	↑: cAMP, Epac1, Rap1 and Rac	↑: sAPPα	[27]
			Mice	Prucalopride 5 and 10 mg/kg, s.c., 90 min ML10302 20 mg/kg, s.c., 90 min	NR	↑: sAPPα	[24]
	Human 5-HT_4(d)_R	Prucalopride (A)	Cell	1 μM, 30 min	NR	↓: Aβ ↑: sAPPα	[29]
	Human 5-HT_4(e)_R	5-HT (A), Prucalopride (A) and Renzapride (A)	Cell	5-HT 1 μM, 30 min Prucalopride 1 μM, 30 min Renzapride 1 μM, 30 min	↑: PKA	↑: sAPPα	[25]
**5-HT_6_R**		ST1936 (A)	Mice	1.3 μM, rev md, 22-24 hours	**Relate**: PKA, MEK and ERK	↓: Aβ	[13]
		EMD-386088 (A) and SB-399885 (AN)	Cell	EMD-386088 10 μM, pre-treated 2 hours SB-399885 10 μM, pre-treated 2 hours	NR	↓: Impaired neurite growth and oxidative stress	[30]
		SB258585 (AN)	Cell	1, 3 and 10 μM, 24 hours	**Relate**: β-arrestin2 and CDK5	↓: Aβ	[31]
		SB271046 (AN)	Mice	10 mg/kg/d, i.p., 5 days	**Relate**: APBA1/2	↓: Aβ, β-secretase, neuroinflammation and γ-secretase ↑: Cognition	[32]
**5-HT_7_R**		AS19 (A)	Rat	1 μg/μl, icv, 30 days	NR	↓: Aβ and neuronal apoptosis ↑: Cognition	[34]
		SB-269970 (IN) and knockdown	Cell and mice	**Cell**: 100nM, 3 days Mice: knockdown 5-HT_7_R	**Relate**: CDK5	↓: Tau ↑: Cognition	[53]

A, agonist; ADAM, a disintegrin and metalloprotease; Akt, protein kinase B; AN, antagonist; APBA1/2, amyloid beta (A4) precursor protein-binding, family A, member 2; Aβ, amyloid-β; BDNF, brain-derived neurotrophic factor; cAMP, cyclic adenosine monophosphate; CaN, calcineurin; CDK5, cyclin-dependent kinase 5; CREB, cAMP-response element binding protein; CTF-α, amyloid precursor protein C-terminal fragment α; C. elegans, caenorhabditis elegans; ERK, extracellular regulated protein kinases; Epac, exchange protein directly activated by cAMP; GR, glucocorticoid receptor; GSK-3β, glycogen synthase kinase-3β; icv, intracerebroventricular injection; i.p., intraperitoneal injection; IN, inverse agonist; MEK, mitogen-activated protein-ERK kinase; MMP-9, metalloproteinase 9; NFAT, nuclear factor of activated T cells; NF-κB, nuclear factor κB; NMDA-Rs, N-methyl-D-aspartate receptors; NR, not reported; PI3K, phosphatidylinositol 3-kinase; PKA, cyclic-AMP dependent protein kinase A; p.o., per os; Rac, Ras-related C3 botulinum toxin substrate; Rap, Ras-related protein; rev md, reverse microdialysis; sAPPα, soluble amino-terminal ectodomain of APP; scip, subcutaneous infusion pump; s.c., subcutaneous injection; TLR2/4, Toll-like receptor 2 and 4; β-arrestin2, a signal-regulating protein. *, effective dose not reported.

#### 2.3.2 Modulation of peripheral inflammation by the 5-HT system

In conclusion, both the central and peripheral 5-HT systems can modulate inflammatory responses. They achieve this by decreasing pro-inflammatory mediators or proteins associated with inflammatory pathways while increasing anti-inflammatory factors. As a result, these actions enhance therapeutic outcomes in AD. Despite extensive evidence supporting the role of the 5-HT system in modulating inflammation, the precise mechanisms of action remain unclear. Further exploration is necessary to support the development of more effective anti-inflammatory drugs and therapies for AD (Table 2).

**Table 2:** Effects of SSRIs and other 5-HTergic enhancement modalities on AD.

Modalitie	Type	Subject	Program	Potential pathway	AD-related pathological changes	Ref.
**SSRIs**	Fluoxetine	Cell	10 μM, 24 hours	NR	↓: Aβ	[31]
		Mice	5 mg/kg/d, p.o., 2 months	NR	↓: Aβ ↑: Cognition	[35]
		Mice	10 mg/kg/d, i.g., 4 months	↑: PP2A, Wnt and β-catenin, ↓: GSK-3β	↓: Aβ, β-secretase and γ-secretase ↑: α-secretase and cognition	[38]
		Mice	5 mg/kg/d, p.o., 4 months	Relate: 5-HT_2_R	↓: Aβ and neurotoxicity	[39]
	Citalopram	Cell	10 μM, 24 hours	NR	↓: Aβ	[31]
		Mice	5 and 10 mg/kg, i.p., 0-24 hours; 8 mg/kg/d, p.o., 4 months	↑: MEK1/2 and ERK	↓: Aβ ↑: α-secretase and CTF-α	[12]
		Mice	10 mg/kg, i.p., 21-24 hours	Relate: PKA	↓: Aβ	[13]
		Mice	10 mg/kg, i.p., 24 hours	NR	↓: Aβ ↑: α-secretase	[36]
	Escitalopram	Cell	80 μM, 24 hours	↓: GSK-3β ↑: Akt	↓: Tau ↑: synaptic protein	[45]
		Rat	10 mg/kg, p.o., 28 days	↓: GSK-3, JNK and c-Jun ↑: PI3K, Akt, Raf-1, MEK, ERK and CREB	↓: Aβ, Tau, β-secretase, neuroinflammation, oxidative stress and CTF-α ↑: α-secretase, BDNF and cognition	[37]
		Rat	10 mg/kg, p.o., 28 days	↓: GSK-3β ↑: Akt	↓: Tau ↑: Cognition	[44]
		Mice	5 mg/kg, i.p., 8 and 24 hours; 2.5 and 5 mg/kg/d, p.o., 4 months; 5 mg/kg/d, i.p., 28 days.	NR	↓: Aβ ↑: α-secretase	[36]
		Mice	10 mg/kg, i.p., 4 weeks	↓: GSK-3β ↑: Akt	↓: Tau and neuronal apoptosis	[48]
	Sertraline	Cell	3 μM, 24 hours	NR	↓: Aβ	[31]
**Kai-Xin-San**	None	Rat	10 g/kg/d, p.o., 28 and 33 days	↓: GSK-3β ↑: Akt and PI3K	↓: Tau and neuroinflammation ↑: ACh and cognition	[47]
**HTrp**	None	Mice	0.40 g TrP/100 g, 30 days	NR	↓: Aβ	[41]

ACh, acetylcholine; Akt, protein kinase B; Aβ, amyloid-β; BDNF, brain-derived neurotrophic factor; c-Jun, a transcription factor protein; CREB, cAMP-response element binding protein; CTF-α, amyloid precursor protein C-terminal fragment α; C. elegans, caenorhabditis elegans; DAF-16, FOXO transcription factor; ERK, extracellular regulated protein kinases; GSK-3β, glycogen synthase kinase-3β; HTrp, high tryptophan diet; i.g., intragastric injection; i.p., intraperitoneal injection; JNK, c-Jun N-terminal kinase; MEK, mitogen-activated protein-ERK kinase; NR, not reported; PI3K, phosphatidylinositol 3-kinase; p.o., per os; PKA, cyclic-AMP dependent protein kinase A; PP2A, protein phosphatases of type 2A; Raf-1, a serine/threonine kinase; SSRIs, selective 5-HT reuptake inhibitors; TrP, L-tryptophan; Wnt, a class of signaling proteins; β-catenin, a class of signaling proteins.

### 2.4 5-HT system synergizes and facilitates with the cholinergic system

The "cholinergic hypothesis" is one of the earliest theories proposed regarding the pathogenesis of AD. It suggests that a severe loss of cholinergic function in the CNS plays a crucial role in AD. This dysfunction may occur before the development of Aβ and Tau pathology [87, 88]. Dysfunction in central or peripheral cholinergic systems leads to neuronal damage in the brain, worsens neuroinflammation, and disrupt the structure and function of brain mitochondria [89-91]. These effects contribute to cognitive impairment in patients with AD [92, 93]. Therefore, improving cholinergic represents an important target for the treatment of AD. Acetylcholine (ACh) plays a crucial role in cholinergic neurotransmission. Researchers are exploring combinations of 5-HTR agonists or antagonists with ACh receptor agonists or acetylcholinesterase inhibitors (AChEIs) as potential treatments for AD. Among these strategies, the 5-HT_3_R, 5-HT_4_R, and 5-HT_6_R subtypes have demonstrated significant effects in various studies.

#### 2.4.1 Multi-target drugs: 5-HT_3_R antagonists and α7 nAChR agonists

A class of multi-targeted drugs, including tropisetron and three novel compounds (RG3487, EVP-6124, and MEM3454), has been studied in both normal and aged rats, as well as elderly rhesus macaques. These drugs can antagonize 5-HT_3_R and can stimulate α7 nicotinic receptors (α7 nAChR). This dual action increases central ACh levels and enhances cognitive performance. They may also provide additional benefits when combined with AChEIs for cognitive function improvement [94-97]. These findings suggest that the compounds may improve cognitive deficits by targeting 5-HT_3_R antagonism and α7 nAChR stimulation. They may also provide additional benefits when combined with AChEIs for cognitive function improvement. However, the clinical impact of these compounds on cognitive function varies [98, 99]. This discrepancy may arise from flaws in experimental design or from a lack of selectivity towards α7 nAChR among this class of drugs [100]. While some researchers emphasize the role of activating α7 nAChR, it is essential to also recognize the positive effects of enhancing cholinergic nerve transmission via 5-HT_3_R antagonism on cognitive deficits [95]. During the process of drug development and clinical trials, overemphasizing the development of highly selective drugs might result in neglect of the therapeutic potential of other effective targets [101]. This perspective is more logical and helps explain why many highly selective drugs often yield unsatisfactory results in clinical trials, possibly due to their limited action on other beneficial targets. Furthermore, there is an urgent need for more clinical studies specifically investigating the cognitive effects of these compounds. Although early-stage research indicates that these compounds may enhance cognitive performance, researchers must conduct large-scale, long-term clinical trials to thoroughly evaluate their safety and efficacy.

#### 2.4.2 5-HTRs

Several 5-HT_4_R agonists, including VRX-03011, SL65.0155, BIMU 1, RS 67333, prucalopride, and PRX-03140, enhance the central cholinergic system and increase the release of ACh in normal rats. This enhancement correlates with improved memory and the reversal of cognitive deficits in the water maze task induced by the muscarinic antagonist scopolamine [102-104]. RS 67333, a specific 5-HT_4_R agonist, shows potential when combined with AChEIs to further augment its memory-enhancing effects in NMRI mice. Notably, this cognitive enhancement can be completely blocked by a 5-HT_4_R antagonist, underscoring the pivotal role of 5-HT_4_R in improving memory deficits [105]. These studies collectively suggest that 5-HT_4_R activation can elevate central ACh levels, restore cholinergic system function, and consequently improve cognitive function. Furthermore, combining 5-HT_4_R agonists with AChEIs appears to enhance these effects, advocating for multi-targeted therapeutic strategies as potentially more efficacious than single-targeted approaches.

Bourson and colleagues were the first to explore the role of 5-HT_6_R in vivo. They demonstrating that antagonism of 5-HT_6_R blocked scopolamine-induced stretching behavior in rats [106], indicating modulation of cholinergic neurotransmission by 5-HT_6_R. Furthermore, 5-HT_6_R antagonism not only enhances cognitive function in normal rats but also ameliorates scopolamine-induced cognitive deficits by enhancing cholinergic neurotransmission [107, 108]. Notably, both 5-HT_6_R antagonists and agonists reversed memory deficits induced by cholinergic damage in rats [109]. Although this seems contradictory, it may be due to the differences in their respective pathways of action. 5-HT_6_R is sparsely present in cholinergic neurons but predominantly found in GABAergic neurons [110]. 5-HT_6_R agonists enhance cholinergic neurotransmission by directly activating the limited number of 5-HT_6_Rs found on cholinergic neurons. 5-HT_6_R antagonists primarily target 5-HT_6_Rs located on inhibitory GABAergic interneurons that are upstream of the cholinergic system. By inhibiting these interneurons, 5-HT_6_R antagonists indirectly promote cholinergic neuronal activity [109]. Moreover, beyond their direct effects on the cholinergic system and cognitive function, 5-HT_6_R antagonists synergize with AChEIs. For instance, the 5-HT_6_R antagonist idalopirdine (10 mg/kg, p.o.) increased extracellular 5-HT levels in the prefrontal cortex of Sprague-Dawley rats and potentiated donepezil-induced increases in ACh [111]. When combined, 5-HT_6_R antagonists and AChEIs activated neural circuits and neurotransmitter systems across multiple brain regions more extensively in awake rats, surpassing the efficacy of single-agent treatments [112]. These positive findings underscore the potential role of 5-HT_6_R in modulating the cholinergic system, leading to the initiation of several 5-HT_6_R antagonist clinical trials.

Despite this, Phase III clinical trials combining 5-HT_6_R antagonists with AChEIs did not produce satisfactory results [113]. However, this does not imply that 5-HT_6_R is irrelevant to AD treatment. On the contrary, 5-HT_6_R antagonists have shown positive results in preclinical studies and Phase II clinical trials. In Phase II trials, these antagonists, when used as adjunctive therapy to AChEIs, led to significant improvements in cognitive function in AD patients. In contrast, Phase III trials did not replicate these findings. This discrepancy may primarily arise from variations in dosage. Additionally, differences in disease severity may also contribute to this discrepancy. For instance, higher doses of idalopirdine (90 mg/d, 30 mg three times daily) and SB-742457 (35 mg/d) used in Phase II trials might have affected cognitive function differently than the lower doses used Phase III trials. Moreover, Phase II studies primarily involved patients with mild-to-moderate AD, and variations in disease progression could have influenced the expression levels of drug targets in vivo, potentially affecting the outcomes (see Table 3). Given the complex and not yet fully understood role of 5-HT_6_R, future research should explore 5-HT_6_R agonists or inverse agonists, both of which have shown potential in AD therapy [109, 114, 115]. Therefore, future studies should carefully consider factors such as dosage, inclusion criteria, and pharmacokinetics.

**Table 3:** 5-HT_6_R antagonists as adjunct to AChEIs on change in cognition in patients with AD.

Compound	Participants	Program	Study (year)	Effect	Ref.
**Idalopirdine**	Mild-moderate AD	1 (28 weeks) A: Idalopirdine (30 and 60 mg/d) + Donepezil (10 mg/d) B: placebo 2 (24 weeks) A: Idalopirdine (30 and 60 mg/d) + Donepezil (10 mg/d) + Memantine B: placebo	Phase III extension study (2019)	No effect	[256]
**Idalopirdine**	Mild-moderate AD	1 (24 weeks) A: Idalopirdine (30 and 60 mg/d) + Donepezil (10 mg/d) B: placebo 2 (24 weeks) A: Idalopirdine (10 and 30 mg/d) + Donepezil (10 mg/d) B: placebo 3 (24 weeks) A: Idalopirdine (60 mg/d) + Donepezil, Rivastigmine, or Galantamine (10 mg/d) B: placebo	Phase III (2018)	No effect	[257]
**Idalopirdine**	Moderate AD	(24 weeks) A: Idalopirdine (90 mg/d, 30 mg thrice daily) + Donepezil (10 mg/d) B: placebo	Phase Ⅱ (2014)	Improvement	[258]
**SB-742457**	Mild-moderate AD	(24 weeks) A: SB-742457 (5 mg/d) B: SB-742457 (15 mg/d) C: SB-742457 (35 mg/d) D: placebo	Phase Ⅱ (2010)	No effect	[259]
**SB-742457**	AD	(12, 24, and 48 weeks) A: SB-742457 (35 mg/d) B: placebo	Phase Ⅱ (2015)	Improvement	[260]

AChEIs, acetylcholinesterase inhibitors; AD, Alzheimer’s disease. *All of the above AChEIs (Donepezil, Rivastigmine, or Galantamine) are stable treatments.

In addition to the previously mentioned 5-HTR subtypes, several other subtypes have received less attention. However, limited evidence indicates that these subtypes play significant roles in regulating the cholinergic system and improving cognitive deficits. For instance, the 5-HT_1A_R antagonist NAD-299 has shown potential in enhancing cognitive performance in Sprague-Dawley rats by enhancing cholinergic function [116]. The 5-HT_2A_R has been associated with central ACh release [117]. The multifaceted 5-HT drug vortioxetine demonstrates promise in modulating various neurotransmitters, including those in the cholinergic system [118]. SSRIs enhance the central cholinergic system through binding to the endoplasmic reticulum protein sigma-1 receptor [119, 120]. Moreover, 5-HT induces peripheral ACh release in a concentration-dependent manner, an effect that may be beneficial in the treatment of AD, despite the limited ability of ACh to cross the BBB [90, 121].

The close relationship between the 5-HT system and the cholinergic system has sparked interest in understanding the mechanisms of their interaction. As previously mentioned, the simultaneous antagonism of the 5-HT_3_R and stimulation of the α7 nAChR, along with activation of the 5-HT_4_R, or activation/antagonism of the 5-HT_6_R can enhance ACh release and mitigate cognitive deficits in rodents. These interactions could also be leveraged in combination with AChEIs to amplify their effects on the cholinergic system. However, to our knowledge, no study has yet fully elucidated the specific pathways through which 5-HTRs influence the cholinergic system and participate in the pathogenesis of AD. Nevertheless, substantial evidence indicates the existence of an interactive relationship between the two systems. Both neurotransmitter systems coexist in several key brain regions (e.g., septal nucleus, medial septum, hippocampus, and striatum, etc.), and interact through specific receptor subtypes. Although in some cases the direct anatomical link is not clear, it is generally possible to detect interactions between 5-HTergic and cholinergic systems using electrophysiological recording techniques [122]. Moreover, evidence from human EEG studies supports these interactions [123]. In Drosophila, researchers have found that ACh released from Kenyon cells activates 5-HTergic dorsal medial paired neurons, demonstrating a bidirectional feedback mechanism between the two systems [124]. These findings provide clues on how the 5-HT and cholinergic systems regulate each other, and the existence of similar mechanisms of action in AD can be explored in detail in the future.

In summary, the 5-HT system has the potential to benefit AD treatment by enhancing the cholinergic system and interacting with ACh to improve cognitive function. However, the clinical efficacy of combining 5-HT and cholinergic drugs may not meet expectations. This discrepancy warrants further research to comprehensively elucidate the specific mechanisms by which the 5-HT system operates in this context and to confirm its clinical impact.

### 2.5 Modulation occurs between the 5-HT system and the BDNF system.

Brain-derived neurotrophic factor (BDNF) is a secreted protein that is crucial for enhancing synaptic plasticity, promoting neurogenesis, and facilitating learning and memory processes [125, 126]. It plays a significant role in alleviating symptoms of AD. Impaired BDNF signaling in AD is associated with Tau hyperphosphorylation, Aβ deposition, neuroinflammation, neuronal apoptosis, and cognitive impairment [126, 127]. Clinical studies consistently demonstrate reduced levels of BDNF in both peripheral and central systems of AD patients [128-130]. Although exogenous BDNF has limited ability to diffuse across the BBB, elevated serum BDNF levels are linked to improvements in cognitive function in AD patients [131]. Therefore, BDNF represents an important therapeutic target for treatment of AD.

#### 2.5.1 5-HT system regulates the BDNF system

Several 5-HTRs have been implicated in central BDNF release. Treatment with the 5-HT_1A_R antagonist NAD-299 and the 5-HT_2A_R agonist TCB-2, either alone (5 μg/μl, respectively) or in combination (5 μg/0.5 μl, respectively), increased hippocampal BDNF levels and ameliorated memory deficits in AD rats [20]. In contrast to AD animal models, activation of 5-HT_1A_R enhances hippocampal BDNF expression in mouse models of depression, cerebral ischemia, and cognitive impairments associated with schizophrenia [132-134]. Additionally, activation and antagonism of 5-HT_6A_R enhance BDNF expression in the hippocampus of non-AD rodents, which is accompanied by improved cognitive function [114, 115, 135]. In a rat stress model, the multi-target 5-HT drug piromelatine (50 mg/kg, i.p.) increased central BDNF expression and ameliorated cognitive dysfunction [136, 137]. Piromelatine (50 mg/kg, i.p.) also exhibited a positive effect in ameliorating cognitive deficits in AD model mice, although changes in BDNF levels were not reported [138]. Collectively, these studies suggest that 5-HT mediates BDNF expression and potentially improves cognitive dysfunction. However, relatively few studies have investigated its role and specific mechanisms in AD models, indicating the need for further research.

Enhancement of the 5-HT system significantly increases both peripheral and central BDNF expression. SSRIs such as fluoxetine, sertraline, paroxetine, and fluvoxamine induce the release of BDNF from platelets, which in turn increases serum concentrations of BDNF [139, 140]. Studies indicate that higher serum BDNF levels correlate with improved cognitive function in AD patients [131]. Additionally, a high Trp diet enhances BDNF expression in the prefrontal cortex and hippocampus of aged rats, suggesting that the 5-HT system may help prevent cognitive dysfunction during normal aging [141]. Further studies support these findings by demonstrating enhanced learning and memory consolidation in animal models [142] (Table 4).

**Table 4:** The 5-HT system affects the cholinergic system and BDNF and is accompanied by an increase in cognitive functioning.

5-HTR, SSRI or multi-target	Subtype	Agent	Subject	Program	Potential pathway	Related pathological changes	Ref.
**5-HT_1_R**	5-HT_1A_R	NAD-99 (AN)	Rat	5 μg/μl, icv, 30 days	NR	↓: Aβ and neuronal apoptosis ↑: BDNF and cognition	[20]
**5-HT_2_R**	5-HT_2A_R	TCB-2 (A)	Rat	5 μg/μl, icv, 30 days	NR	↓: Aβ and neuronal loss ↑: BDNF and cognition	[20]
**5-HT_4_R**		Prucalopride (A) and PRX-03140 (A)	Cell and rat	**Prucalopride** 5, 5.6 and 10 mg/kg, s.c. 3 or 4 hours **PRX-03140** 5.6 and 50 mg/kg, s.c., 3 or 4 hours	NR	↑: ACh, cAMP and stimulated-hippocampal θ power	[103]
		VRX-03011 (A)	Rat	1, 5 and 10 mg/kg, i.p., 30 min	NR	↑: ACh and cognition	[102]
		SL65.0155 (A)	Mice	0.1 and 0.3 mg/kg, i.p., 4 days; 0.001 - 0.1 mg/kg, p.o. and i.p., 24 hours; 0.01 and 0.1 mg/kg, i.p., 10 days	NR	↓: Cognitive impairment (Induced by cholinergic dysfunction)	[104]
**5-HT_6_R**		WAY-181187 (A)	Rat	3 mg/kg, i.p., 5 days	NR	↓: Dendritic injury ↑: BDNF and cognition	[115]
		WAY208466 (A)	Mice	9 mg/kg, i.p., 30 min	↑: MEK, ERK1/2	↑: BDNF and cognition	[114]
		E-6801 (A) and EMD 386088 (A)	Rat	**E-6801** 2.5 and 5 mg/kg, i.p., 30 min **EMD 386088** 5 and 10 mg/kg, i.p., 30 min	NR	↓: Cognitive impairment (Induced by cholinergic dysfunction)	[109]
		WAY-181187 (A) and SB-742457 (AN)	Rat	**WAY-181187** 1 and 3 mg/kg, i.p., 60 min or 21 days; **SB-742457** 1 and 3 mg/kg, i.p., 60 min or 21 days	NR	↑: BDNF and cognition	[135]
		SB-399885 (AN)	Rat	10 mg/kg, p.o., twice a day for 7 days	NR	↓: Cognitive impairment (Induced by cholinergic dysfunction) ↑: ACh and cognition	[107]
		RO4368554 (AN)	Rat	1, 3, 10 and 30 mg/kg, i.p., 100 mg/kg, p.o., Immediate administration or 60 min;	NR	↓: Cognitive impairment (Induced by cholinergic dysfunction) ↑: Cognition	[108]
		Idalopirdine (AN)	Rat	2 mg/kg, i.v., 60 min	NR	↑: Cholinergic system	[112]
**SSRIs**		Fluoxetine	Mice	10 mg/kg, i.p., twice a day for 15 days^&^	↑: CREB	↑: BDNF, synaptic proteins, number of neurons, dendritic spine density, synaptic plasticity, and cognition	[146]
			Mice	10 mg/kg, i.p., twice a day for 15 days (check 20 days after last injection)	↑: CREB	↓: Aβ ↑: Synaptic plasticity, number of neurons, dendritic spine density, cognition	[147]
**Multi-target**		YL-0919 (*)	Rat	1.25 and 2.5 mg/kg, i.g., 20 days	NR	↑: BDNF, synaptic proteins, dendritic complexity, spine density and cognition.	[136]
		Piromelatine (#)	Rat	50 mg/kg/d, i.p., 2 weeks	↑: CREB	↑: BDNF, hippocampal cytogenesis deficit and cognition	[137]

A, agonist; ACh, acetylcholine; AN, antagonist; Aβ, amyloid-β; BDNF, brain-derived neurotrophic factor; cAMP, cyclic adenosine monophosphate; CREB, cAMP-response element binding protein; ERK1/2, extracellular regulated protein kinases 1/2; icv, intracerebroventricular injection; i.g., intragastric injection; i.p., intraperitoneal injection; i.v., intravenous; MEK, mitogen-activated protein-ERK kinase; NR, not reported; p.o., per os; SSRIs, selective 5-HT reuptake inhibitors; s.c., subcutaneous injection; &, fluoxetine was applied through peritoneal injection to the mice at postnatal day 35 for 15 consecutive days, and the effects of fluoxetine were observed at 6-month; *, A novel combined selective 5-HT reuptake inhibitor/5-HT_1A_R partial agonist/5-HT_6_R full agonist; #, A melatonin and 5-HT_1A_R and 5-HT_1D_R agonist.

CREB is known to play an important role in neuroprotection and is able to regulate specific target genes such as BDNF and tropomyosin receptor kinase B (TrkB) [143]. The signaling pathways of CREB and BDNF are tightly coupled. When CREB is phosphorylated, it increases BDNF expression, which in turn activates TrkB and subsequently induces CREB activation. SSRIs can exert neuroprotective effects by up-regulating CREB expression, increasing BDNF levels, and promoting the expression of TrkB, the primary receptor for BDNF [144, 145]. In adolescent 3×TgAD mice treated with fluoxetine (10 mg/kg, i.p.) twice daily for 16 days, up-regulation of hippocampal and cortical CREB/BDNF signaling pathways was observed during adulthood. Additionally, there was an increase in the levels of synapse-associated proteins in the hippocampus and cortex, the neuron numbers, dendritic spine densities, and a remodelling of synaptic plasticity in hippocampal neurons. These changes ultimately manifested as improvement in brain atrophy and cognitive deficits in the adult mice [146]. The above changes were similarly observed in another 3×TgAD mice study and were accompanied by an improvement in Aβ pathology [147]. 5-HT has been found to activate CREB and promote BDNF release through multiple pathways: (i) PI3K/Akt pathway: After fluvoxamine treatment, mRNA and protein levels of BDNF, CREB, and Akt were significantly elevated in peripheral mononuclear cells of patients. This elevation indicates a positive correlation among the expression levels of CREB, Akt, and BDNF [148]. This suggests that SSRIs may activate the Akt/CREB pathway, thereby enhancing BDNF expression. (ii) Calmodulin-dependent protein kinase IV (CaMK IV) and mitogen-activated protein kinase (MAPK): Fluoxetine is involved in CREB phosphorylation by increasing the enzymatic activity of nuclear Ca^2+^/CaMK IV and the expression levels of the MAP kinases ERK1/2 in the prefrontal/frontal cortex (PFCX) of Sprague-Dawley rats [149], which in turn may indirectly enhance BDNF expression. (iii) TrkB/phospholipase-Cγ1 (PLCγ1) pathway: Fluoxetine (30 mg/kg, i.p.) induces TrkB phosphorylation in the mice anterior cingulate cortex. This activation subsequently triggers PLCγ1, leading to an increase in CREB phosphorylation [144]. Interestingly, the rapid TrkB activation induced by citalopram in the anterior cingulate cortex of the mice was hindered when 5-HT levels were reduced in the mouse brain using pCPA. This suggests that SSRIs enhance BDNF expression through the TrkB/PLCγ1/CREB signaling pathway, likely mediated by increased levels of 5-HT. In addition, 5-HT may enhance BDNF release by activating the PLC pathway via 5-HT_2A_R and 5-HT_6_R in NG2 cells [150]. These studies indicate that the 5-HT system potentially influences both central and peripheral BDNF expression through the activation of CREB and PLC-related pathways. However, certain pathways remain uninvestigated in AD models, necessitating further studies to assess their mechanistic effectiveness in the future.

#### 2.5.2 Interaction between 5-HT and BDNF

The 5-HT system interacts with BDNF, thereby affecting synaptic plasticity and neuronal function [151-153]. BDNF exerts a trophic effect on 5-HT neurons and enhances 5-HT synthesis following partial depolarization of the membrane in raphe nucleus neurons [154, 155]. At the genetic level, the interaction between SERT and BDNF gene polymorphisms correlates with anxiety in healthy individuals [156]. This interaction highlights the effects of SERT and BDNF across various regions of the nervous system and offers insights into potential treatments for neurological diseases and neuroprosthetic strategies. In animal experiments, interactions within the 5-HT-BDNF-NGFR axis among neurons and neuroglia stimulated regenerative neurogenesis in the brains of adult AD zebrafish, underscoring the crucial role of the interaction between 5-HT and BDNF in neural regeneration [157]. Furthermore, BDNF activates G proteins in hippocampal cultures in response to 5-HT_1A_R, demonstrating BDNF modulation of 5-HT neural signaling and its potential implications for neuronal function and cellular signaling. Additionally, experiments with mice modeled of AD or 5-HTergic and noradrenergic degeneration have shown a depletion of 5-HT in critical areas involved in learning and memory, particularly in the hippocampus. This depletion coincided with an increase in BDNF protein levels [158], suggesting a compensatory mechanism in response to the neurodegeneration induced by the loss of 5-HT and norepinephrine. These findings imply that BDNF may protect neurons from neurological damage through compensatory mechanisms. Overall, these studies underscore the pivotal role of 5-HT in modulating BDNF function, although further validation is necessary to elucidate the exact mechanisms underlying their interaction. This complex interplay influences various facets of neural regeneration, repair, mood regulation, and cognitive enhancement, demonstrating an active role in neuroprotection and regeneration in models of AD and other cognitive deficits.

**Figure 1.**
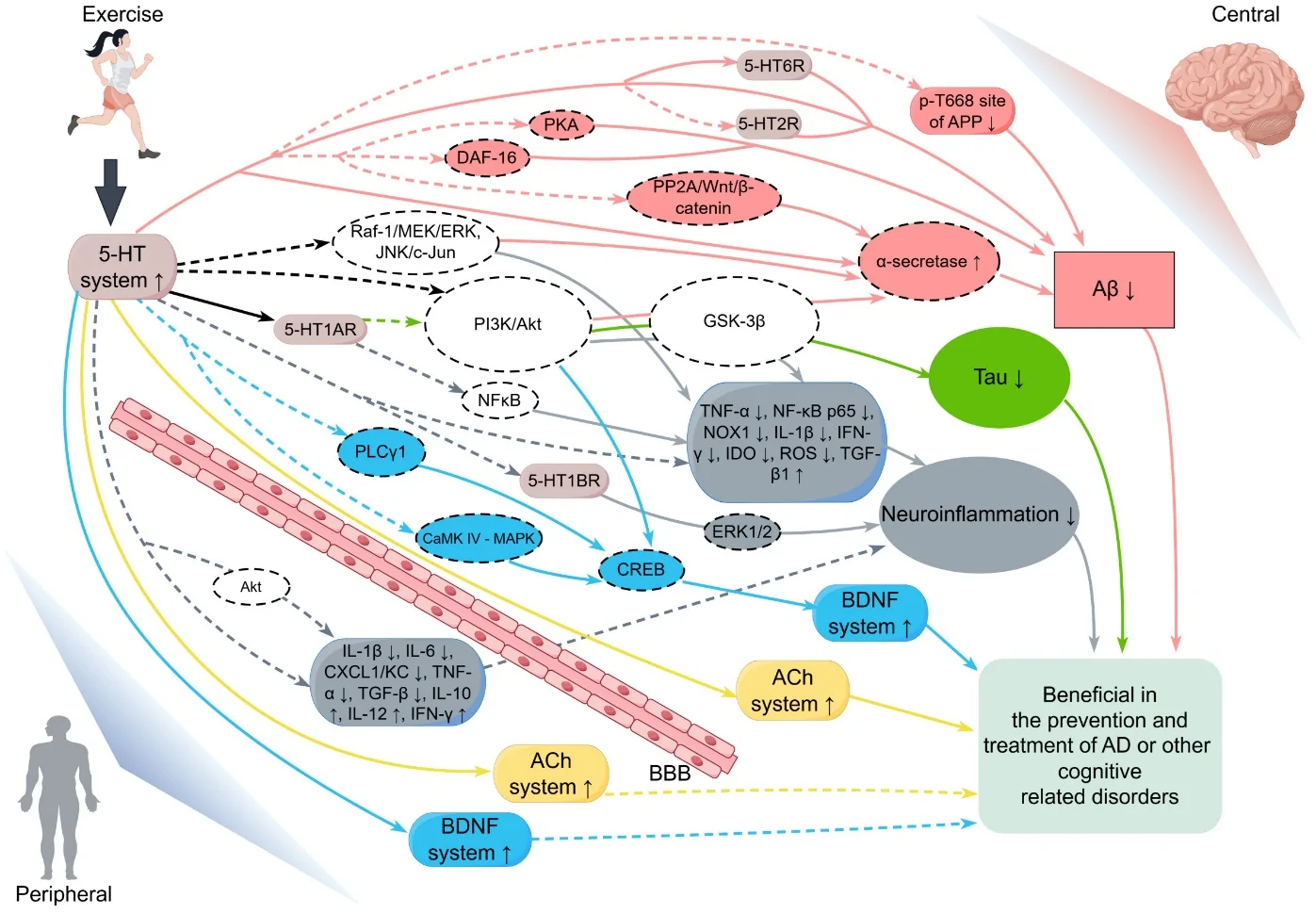
**Schematic representation of potential mechanisms by which the 5-HT system mediates exercise to ameliorate AD pathology**. During exercise, the central and peripheral 5-HT systems ameliorate AD or other cognitive deficits through different pathways (indicated by different colours, where black involves multiple pathways), including proven (shown by solid line) and potential mechanisms (shown by dashed). ACh, acetylcholine; Akt, protein kinase B; APP, amyloid Precursor Protein; Aβ, amyloid-β; BDNF, brain-derived neurotrophic factor; CaMK IV, calmodulin-dependent protein kinase IV; CREB, cAMP-response element binding protein; c-Jun, a transcription factor protein; CXCL1/KC, C-X-C motif chemokine ligand 1; DAF-16, FOXO transcription factor; ERK, extracellular regulated protein kinases; GSK-3β, glycogen synthase kinase-3β; IDO, indoleamine 2,3-dioxygenase; IFN-γ, interferon-γ; IL-10, interleukin-10; IL-12, interleukin-12; IL-1β, interleukin-1β; IL-6, interleukin-6; JNK, c-Jun N-terminal kinase; MAPK, mitogen-activated protein kinase; MEK, mitogen-activated protein-ERK kinase; NF-κB, nuclear factor κB; NOX1, NADPH Oxidase 1; PI3K, phosphatidylinositol 3-kinase; PKA, cyclic-AMP dependent protein kinase A; PLCγ1, phospholipase-Cγ1; PP2A, protein phosphatases of type 2A; Raf-1, a serine/threonine kinase; ROS, reactive oxygen species; TGF-β, transforming growth factor-β; TNF-α, tumor necrosis factor-α; β-catenin, a class of signaling proteins. For details, please see the main text.

The above studies suggest that the 5-HT system promotes the release of BDNF in both central and peripheral pathways. Moreover, the interaction between 5-HT and BDNF is crucial for the health of the nervous system. Despite an incomplete understanding of the mechanisms underlying these pathways, the intricate relationship between 5-HT and BDNF offers significant insights and avenues for studying neurological health mechanisms, including disorders such as AD. In conclusion, the 5-HT system significantly contributes to neuroprotection in the brain through the regulation of BDNF (Fig. 1).

## 3. Potential mechanisms of the 5-HT system in AD and exercise interventions

In recent years, exercise has garnered significant attention and research due to its demonstrated positive effects in the treatment of various diseases. Specifically, in the context of AD, exercise has been shown to effectively improve a range of neuropathological features and cognitive dysfunction [14]. However, the precise mechanisms of its action remain incompletely understood. As a neurotransmitter sensitive to movement, 5-HT is significantly influenced by exercise. Different modes of exercise can enhance both central and peripheral 5-HT systems; however, the extent of this enhancement depends on various factors, including exercise intensity, duration, and the specific effects on different brain regions [159-163]. Furthermore, several studies have highlighted the role of the 5-HT system in exercise-induced neurogenesis and memory improvement, both of which are beneficial for AD [15-17]. This suggests that the 5-HT system plays an important role in how exercise improves AD. However, no studies have systematically reviewed the mechanisms through which 5-HT-mediated exercise improves AD pathology, including Aβ deposition, Tau hyperphosphorylation, neuroinflammation, and cholinergic dysfunction. Given the similarities between the effects of exercise and SSRIs on the 5-HT system, it is reasonable to hypothesize that the mechanisms through which SSRIs ameliorate AD pathology may partly represent the potential mechanism of 5-HT system-mediated exercise in improving AD. Therefore, this section explores the potential mechanisms through which 5-HT system-mediated exercise in improving AD, drawing on insights from how exercise modulates the 5-HT system and how SSRIs ameliorate AD.

### 3.1 Potential mechanisms of 5-HT in exercise-induced improvement of AD

#### 3.1.1 5-HT system mediates exercise regulation of Aβ pathology

Firstly, exercise modulates Aβ production through 5-HT-mediated APP cleavage. Yoga and meditative exercises activate the α-secretase pathway of APP cleavage via 5-HT, resulting in the production of the fragment sAPPα, which is known to have neurotrophic effects and, in turn, inhibits oligomeric Aβ production [163]. Secondly, exercise enhances levels of the 5-HT system, which ultimately exerts a lowering effect on Aβ levels through direct action on Aβ protofibrils. Four weeks of incremental treadmill exercise (10-60 min per day) increased the number of 5-HTergic neurons in the raphe nucleus of aged APP/PS1 mice. This regimen significantly reduced hippocampal Aβ_1-40_ and Aβ_1-42_ the deposition, and improved spatial learning and memory [164]. This may be due to the fact that 5-HT and melatonin ultimately regulate Aβ levels by disrupting the local β-sheet structure and the salt bridge between the K28 side chain and A42 COO^-^, or by π-π binding to N-terminal aromatic residues and C-terminal hydrophobic residues, which in turn disrupts the overall structure and stability of the Aβ protofibrils [165]. Furthermore, the administration of treadmill exercise (15-45 min, three times per week) for a period of 32 weeks enhanced 5-HT levels and 5-HT_6_R expression in the hippocampus and cortex of AD rats while simultaneously attenuating Aβ pathology [166]. These studies suggest that exercise may target the 5-HT system to improve Aβ pathology in AD through multiple pathways. In addition, due to the similarity in how both exercise and SSRIs affect the 5-HT system, SSRIs also improve Aβ pathology through various pathways. These pathways include inhibiting the phosphorylation of APP at the T668 site, enhancing the α-secretase cleavage of APP, or acting through DAF-16 and PKA-related pathways. Overall, these mechanisms suggest potential ways through which 5-HT-mediated exercise ameliorates Aβ pathology. Future studies could delve deeper into the regulatory processes of these mechanisms and explore their specific applications in clinical therapy.

#### 3.1.2 5-HT system involvement in exercise for ameliorating Tau pathology

Although direct studies on the effects of exercise on Tau pathology via the 5-HT system are preliminary and lack conclusive evidence, clues related to the 5-HT system can still be pursued in studies investigating the link between exercise and Tau phosphorylation. The intervention model of an enriched environment (EE), which includes physical activities such as running on a wheel and cognitive stimulation, has been found to increase 5-HT turnover in the hippocampus of adult rats of both sexes. Notably, female rats exhibited lower levels of Tau phosphorylation in the hippocampus compared to male rats, indicating sex-specific effects of this intervention model on the regulation of Tau phosphorylation. Interestingly, this model suppressed the expression of hippocampal 5-HT_1A_R and led to higher levels of Tau phosphorylation in male rats. Despite these effects, male rats subjected to the intervention unexpectedly demonstrated improved object recognition memory and exploratory behavior compared to the control group [167]. In contrast, previous studies have shown that running wheel exercise increases the expression of 5-HT_1A_R and thus decreases Tau phosphorylation [14, 168]. The inconsistent results may be attributed to several factors. Firstly, the EE intervention model, which includes physical activity, is not solely an exercise intervention. Secondly, the sex-specific nature of this intervention in regulating Tau phosphorylation regulation could also play a role. Furthermore, the suppression of 5-HT_1A_R expression in the rat hippocampus may explain these findings. Based on the finding that activation of 5-HT_1A_R reduces Tau phosphorylation levels in the hippocampus of rats via the PI3K/Akt/GSK-3β [45], it is speculated that this intervention may have blocked the regulation of Tau phosphorylation through this pathway by inhibiting 5-HT_1A_R. In addition, SSRIs can modulate Tau pathology by activating the Akt/GSK-3β pathway in a non-5-HT_1A_R-dependent manner. This potential mechanism, through which 5-HT-mediated exercise may ameliorate Tau pathology, needs verification in further experiments. Overall, although the exact relationship between Tau phosphorylation and 5-HT_1A_R, along with its mechanism of action, requires further exploration in future studies, current findings suggest that exercise may positively impact Tau pathology through the Akt/GSK-3β signaling pathway.

#### 3.1.3 5-HT system mediates exercise regulation of inflammatory responses

Different exercise modes can affect both central and peripheral inflammatory responses by modulating either the central or peripheral 5-HT system. For central system, four weeks of swimming exercise (60 min, five times a week) elevates 5-HT concentrations in the prefrontal cortex of chronically unpredictable mild stress (CUMS) rats. This exercise significantly inhibits the CUMS-induced increases in pro-inflammatory factors such as TNF-α, interferon-γ (IFN-γ), and IDO in the prefrontal cortex of rats [169]. This would have an inhibitory effect on the kynurenine metabolic pathway of Trp, which in turn prevents neuroinflammation induced by kynurenine metabolite deposition, synaptic dysfunction, and affects neuronal regeneration and degeneration [68]. Moreover, two subtypes of the 5-HT_1_R are play a crucial role in mediating the exercise-induced improvement of central inflammation. Specifically, treadmill exercise exerts varying effects on central inflammation through modulation of 5-HT_1A_R. In Sprague-Dawley rats, four weeks of treadmill exercise (60 min, five times a week) reduces the expression levels of 5-HT_1A_R [170]. Additionally, antagonism of 5-HT_1A_R decreases the levels of microglia and astrocytes in the hippocampus of AD mice and reduces the number of TNF-α positive cells. This may be achieved through crosstalk with the NF-κB signaling pathway [71]. Conversely, in aged rats, 64 weeks of treadmill exercise (30 min three times a week) increased the expression levels of 5-HT_1A_R in both the hippocampus and cortex. This enhancement correlates with increased 5-HT concentrations from the raphe nuclei and improves both spatial and non-spatial memory capacity [168]. This suggests that the effect of exercise on 5-HT_1A_R may vary based on exercise duration and specific brain regions. Studies on another subtype, the 5-HT_1B_R, indicate that two weeks of treadmill exercise (30 min, five times a week) increase both 5-HT levels and its metabolite 5-hydroxyindoleacetic acid (5-HIAA) in the brainstem of mice. Furthermore, this exercise regimen reduces SERT expression while enhancing 5-HT_1B_R expression [171]. Activation of 5-HT_1B_R has shown anti-inflammatory effects and can mitigate AD pathology, potentially through ERK1/2 activation in both cellular and animal models [72]. Furthermore, this exercise regimen shares similarities with SSRIs in modulating the 5-HT system, including SERT inhibition and increased 5-HT levels, suggesting overlapping mechanisms. A study with the SSRI escitalopram indicates that 5-HT may attenuate inflammation by activating pro-survival pathways such as PI3K/Akt/GSK-3β and Raf-1/MEK/ERK while inhibiting pro-apoptotic pathways like JNK/c-Jun [37]. These findings provide valuable insights for further investigating how 5-HT mediates exercise-induced improvements in neuroinflammation.

For the peripheral system, a 12-week regimen of regular yoga practice (90 min, once a week) significantly elevated human plasma 5-HT levels and increased serum immune-related cytokines (IL-12 and IFN-γ) [172]. Notably, exercise impacts central and peripheral levels of IFN-γ differently. This difference arises because CUMS induces excessive elevation of IFN-γ in the rat CNS, whereas exercise reduces IFN-γ levels via 5-HT, thereby exerting an anti-inflammatory effect. In peripheral tissues, exercise induces a balanced increase in the immune cytokine IFN-γ, which interacts with inflammatory processes. Together, these factors help regulate and maintain immune homeostasis in the body. Thus, exercise plays an immunomodulatory role in all these processes. In animal studies, chronic aerobic training reduces the levels of pro-inflammatory factors (IL-1β, IL-6, CXCL1/KC, TNF-α, and TGF-β) in bronchoalveolar lavage fluid and serum by correcting abnormal 5-HT/Akt signaling in lung-injured mice. Additionally, it elevates the level of the anti-inflammatory factor IL-10, thereby ultimately ameliorating the peripheral inflammatory response in mice [173].

In summary, exercise can modulate both central and peripheral inflammatory responses through the 5-HT system. However, research on the effects of exercise in AD models is limited, and the mechanisms involved are not well understood. Furthermore, discrepancies in the exercise-induced modulation of the 5-HT system across previous studies may arise from variations in exercise intensity and the brain regions being investigated [170, 174-176]. Future studies should focus on systematically exploring how exercise affects the 5-HT system in various brain regions of AD models and elucidating the mechanisms by which exercise mediates inflammation reduction.

#### 3.1.4 Exercise promotes interactions between 5-HT and ACh

The interaction between 5-HT and ACh plays a crucial mediating role in behavioral performance, including cognitive functions [177]. Exercise further enhances the interaction by increasing the levels of both neurotransmitters. Studies have demonstrated that various exercise modalities enhance the release of 5-HT and ACh in both the central and peripheral systems. This enhancement positively affects treatment outcomes for AD. In a human intervention study, a 12-week high-intensity interval training program (45 min, three times a week) significantly increased serum concentrations of 5-HT and ACh in older adults. This increase was associated with a significant improvement in executive functions, evidenced by a reduction in choice reaction times [178]. Similarly, in animal studies, even simple walking activities (e.g., a walking speed of 2.3 meters per minute for 5 minutes) were found to increase the release of 5-HT and ACh in the extracellular space of the cerebral cortex of mice [179]. Further studies revealed that platform running exercises upregulated the levels of 5-HT and ACh in the brain of normal and AD model rats and significantly improved cognitive dysfunction in these rats [168, 180]. Additionally, SSRIs can enhance central cholinergic function by increasing the concentration of 5-HT, which in turn promotes the interaction of 5-HT with sigma-1 receptors in the brain [119, 120]. Given the similarities between the effects of exercise and SSRIs on the 5-HT system, it is reasonable to speculate that exercise may positively influence the cholinergic system through analogous pathways. In summary, exercise increases the levels of 5-HT and ACh in both central and peripheral systems, potentially enhancing their interaction and thereby benefiting cognitive function.

#### 3.1.5 5-HT system mediates exercise modulation of the BDNF system

Exercise plays a crucial role in promoting neurological health, especially by enhancing the 5-HT system and the BDNF system and promoting their synergistic effects. A study involving humans found that 12 weeks of high-intensity interval training (45 min, three times a week) elevated serum levels of 5-HT, BDNF, ACh, and various trophic factors in overweight and obese older adults. This indicates potential neuroprotective benefits [178]. In animal experiments, 5-HT plays a potential role in regulating the BDNF system through exercise. Specifically, 64 weeks of aerobic exercise (treadmill exercise, 30 min, three times a week) improved cognitive function and protected the brain from the harmful effects of sedentary behavior and aging. This protection was achieved by increasing levels of 5-HT and BDNF, as well as enhancing the expression of SERT and 5-HT_1A_R in the cortex and hippocampus of aged rats. These changes indicate an enhanced interaction between 5-HT and BDNF. Notably, the enhanced expression of 5-HT_1A_R and SERT correlates with increased 5-HT concentrations in the raphe nuclei due to exercise [168]. Additionally, in stress-induced rats, four weeks of aerobic exercise (treadmill exercise, 30 min, once a day) also increased 5-HT_1A_R expression and was accompanied by enhanced expression levels of BDNF, TrkB, and pCREB in the hippocampus [181]. The above studies suggest that exercise-induced increases in BDNF and TrkB expression may be related to elevated 5-HT concentration and the subsequent activation of 5-HT_1A_R. However, further exploration is needed to confirm the feasibility of this pathway in models of AD or cognitive impairment.

Furthermore, a study involving Trp hydroxylase 2-deficient mice, which lack brain 5-HT, found that the baseline levels of hippocampal neurogenesis in these mice were normal. However, exercise-induced neurogenesis was impaired [16], indicating that central 5-HT release is necessary for this process. Previous research has highlighted the significant roles of both 5-HT_3_R and functional SERT in exercise-induced hippocampal neurogenesis [15, 182]. Given the close association between BDNF and neurogenesis, future studies can explore whether BDNF is involved in this process and elucidate the specific mechanisms involved.

### 3.2 Effects of exercise on the 5-HT system

The changes in 5-HT metabolism in the brain are time-dependent and vary by brain region. In trained rats, the levels of 5-HT and 5-HIAA in the striatum, hippocampus, and midbrain increase following training or acute exercise [160, 183, 184].

Acute swimming exercise (1 hour) significantly increased the synthesis and metabolism of 5-HT in the brainstem and hypothalamus of rats. However, no changes were observed in the cerebral cortex and hippocampus during the acute exercise. In contrast, chronic swimming exercise (for 4 weeks, 6 days per week) enhanced the synthesis and metabolism of 5-HT in the cerebral cortex of rats. Notably, this neuronal adaptation persisted even one week after the cessation of training. Immediately after the completion of training, the turnover rate of 5-HT in the brainstem increased. A delayed effect was observed in the hippocampus, with no immediate change in 5-HT levels following training; however, a subsequent decrease in turnover was noted after one week of rest. In the hypothalamus, 5-HT and 5-HIAA levels decrease immediately following training, and then rebound to increase after a week of rest following training [160]. Furthermore, another experiment involving swimming intervention in rats also found an increase in the level of 5-HT in the brain [185].

Prolonged running exercise results in an increased levels of Trp, 5-HT and 5-HIAA in the midbrain, striatum, and hippocampus of rat. However, the accumulation of 5-HTP varies among different brain regions. A 90-minute running session can increase the accumulation of 5-HTP in the midbrain of rats (following administration of an aromatic amino acid decarboxylase inhibitor) and reduce this accumulation in the hippocampus, while no change is observed in the striatum [183]. This exercise regimen also increased 5-HT levels in the hippocampus and cortex of rats, with the highest levels observed 30 minutes prior to recovery. Subsequently, cortical 5-HT levels exhibited a rapid and significant decline, despite hippocampal levels remaining at their peak [161]. These findings indicate that prolonged exercise generally increases levels of Trp, 5-HT, and 5-HIAA in the three brain regions. However, there are differences in the utilization of Trp in the 5-HT synthesis pathway, as well as in the synthesis and metabolism of 5-HT in different brain regions, leading to variations in the accumulation of 5-HTP across these regions. This also indicates that vigorous exercise induces a delayed increase in the release of 5-HT in the brain, but the recovery shows a brain region-dependent pattern. Furthermore, regardless of whether it is forced treadmill and running wheel exercise or voluntary running wheel exercise, after four weeks of intervention, hippocampal 5-HT levels in vascular dementia rats increased, accompanied by improvements in cognitive function [186, 187]. In AD model mice, 4 weeks of incremental treadmill exercise increased the number of 5-HTergic neurons in the raphe nucleus of mice, significantly reduced the deposition of hippocampal Aβ_1-40_ and Aβ_1-42_, and improved the spatial learning and memory of mice [164].

In the peripheral, whether it is 1, 2, or 3 hours of treadmill exercise, it leads to an increase in free Trp in the plasma of rats and has little effect on the plasma concentrations of other large neutral amino acids that compete with Trp for entry into the brain, thereby increasing the turnover rate of 5-HT in the brain. The level of Trp in the liver only increases after 3 hours of exercise [188, 189]. In human studies, 12 weeks of regular yoga practice (90 min, once a week) or high-intensity intermittent training (45 min, three times a week) significantly increase the 5-HT levels in human plasma and serum, accompanied by an increase in immune cytokines in serum and improvement in executive function [172, 178].

Existing research indicates that, in addition to 5-HT and its metabolite 5-HIAA, moderate-intensity exercise of various durations regulates the expression of 5-HTRs in the brain. For instance, a 2-week treadmill exercise regimen (30 min, five times a week) increases the levels of 5-HT and 5-HIAA in the brainstem, reduces the expression of SERT, and increases the expression of 5-HT_1B_R [171]. Additionally, an 8-week running wheel exercise regimen (60 min, five times a week) promotes hippocampal neurogenesis, mood improvement, memory enhancement, and exerts an antidepressant effect by stimulating 5-HT_3_R [15]. Longer training programs, such as a 32-week treadmill exercise regimen (15-45 min, three times a week), enhance the levels of 5-HT and the expression of 5-HT_6_R in the hippocampus and cortex of AD mice, while also mitigating Aβ pathology [166]. Similarly, a 64-week treadmill exercise (30 min, three times a week) increases the level of 5-HT in the brain of elderly rats and the expression of 5-HT_1A_R in the cortex and hippocampus, thereby improving cognitive function [168]. This suggests that longer cycles of regular exercise may have a beneficial effect on cognitive function. However, the current research on these receptors in AD models is still in the preliminary stage. In the future, it is necessary to further explore the effects of various exercise methods, durations, and intensities on 5-HTRs in different brain regions in AD models.

In summary, the influence of exercise on the 5-HT system significantly demonstrates brain region specificity. This indicates that different brain regions may respond variably to the regulation of the 5-HT system in response to exercise. Since there are relatively few studies related to the improvement of AD pathological mechanisms through exercise via the 5-HT system, we can explore the potential role of the 5-HT system in brain health by examining the specific effects of exercise on the 5-HT system in different brain regions. Future studies can further investigate how various exercise interventions affect cognitive functions through the 5-HT system in different brain regions. For example, researchers could study how different types of exercise (such as aerobic exercise, strength training, or high-intensity interval training) influence cognitive functions such as memory, learning, and emotion through their specific effects on the 5-HT system. Additionally, exploring the changes in the mechanisms of the 5-HT system in these brain regions, as well as the specific effects of exercise on 5-HT synthesis, metabolism, and neural transmission, will help to deeply understand the potential benefits of exercise on brain health and cognitive functions. These studies can not only reveal the complex effects of exercise on brain functions but may also provide a scientific basis for formulating personalized exercise intervention programs to optimize cognitive health and mental state.

## 4. Exercise and medication in AD

Exercise, as a non-pharmacological treatment method, has garnered significant attention due to its promising effects in managing AD. Yu et al. conducted a comprehensive analysis of 11 intervention measures for AD and found that the benefits of physical exercise significantly outweigh its risks. They highlighted that physical exercise holds substantial promise for the treatment of AD and recommended that individuals aged 65 and older engage in regular physical activity [190]. In addition to enhancing cognitive function, exercise can improve the mental well-being and activities of daily living of AD patients. However, it is noteworthy that exercise interventions lasting 16 weeks or less show limited improvement [191, 192]. This indicates that the benefits of exercise for AD may only become apparent with longer-term engagement. Some studies have reported that exercise may not significantly improve cognitive and behavioral deficits in dementia patients [193, 194]. Nevertheless, in the absence of exercise, cognitive function tends to deteriorate more rapidly. In other words, exercise plays a positive role in maintaining cognitive function in dementia patients. For instance, individuals who exercise more than three times a week have only half the risk of developing dementia compared to those who do not exercise [195]. Additionally, a randomized controlled trial (RCT) demonstrated that both group and home exercise interventions significantly reduced the rate of decline in physical function after one year compared to conventional medical treatments [196]. Furthermore, exercise interventions have been found to have low side effects and high adherence, making them easier to incorporate into home care and daily routines.

To date, the AChEIs that have been approved for marketing and used in the treatment of AD include donepezil, galantamine, rivastigmine, and tacrine. Considering the advantages and disadvantages of exercise interventions compared to pharmacological treatments for AD may provide a more comprehensive perspective on its treatment. A meta-analysis comparing the effects of exercise and medication found moderate improvements in cognitive function for both AD and MCI patients in the exercise group. There was a significant improvement in cognitive performance among AD patients taking donepezil, whereas there was no improvement was observed in cognitive performance among MCI patients. Additionally, the effect of exercise on cognition may be more significant than that of donepezil in the AD group [198]. This is consistent with previous research, which suggest that donepezil has relatively limited effectiveness in MCI patients [199, 200]. Exercise has a more beneficial therapeutic effect on MCI patients, consistent with previous meta-analysis [201]. Furthermore, some studies have shown that memantine provides clinical benefits in moderate to severe AD but not in mild AD [202]. Another study found that AChEIs, memantine, and Ginkgo biloba are beneficial for treating AD patients but not for MCI patients [203]. These studies indicate that drug treatment may provide significant benefits for patients with more severe cognitive impairments, while exercise offers greater advantages for those with mild cognitive impairments. This suggests that for patients with cognitive impairments at different stages of pathological progression, various intervention methods can be employed, such as exercise therapy for mild cognitive impairment and a combination of exercise and drug therapy for more severe patients.

Current drug treatments for AD, including memantine and AChEIs, primarily focus on alleviating pathological symptoms. However, they have limited efficacy in addressing associated neuropsychiatric complications, such as anxiety and depression. In contrast, SSRIs have shown promising results. They not only target traditional pathological features of AD, such as Aβ plaques, Tau protein abnormalities, and neuroinflammation, but also alleviate anxiety and depression commonly experienced by AD patients [36-38, 44, 204-206]. In a comparative study, researchers analyzed brain Aβ deposition in participants who had not received antidepressant medication within the past five years compared to those who had. They found that the increased Aβ levels in untreated participants occurred in the same brain regions previously identified as being affected in AD [12]. This finding suggests that antidepressant medication may be associated with a reduction or modulation of Aβ deposition, which in turn may influence the progression of AD.

Existing studies have demonstrated that SSRIs are safe, consistent, and effective at the doses utilized in preclinical trials. It is noteworthy that there is a degree of conversion between doses in mice and humans. For example, the dose of citalopram is 10 mg/kg in mice, which corresponds to 50 mg/day in humans [207]. In contrast, escitalopram is administered at 5 mg/kg/d in mice, which corresponds to a dose of approximately 24 mg for a 60 kg individual. This is comparable to the typical prescribed doses of 10 mg and 20 mg for humans [208]. The relative consistency of these doses suggests that SSRIs are comparable across species and can serve as a reliable dosage reference for clinical applications. Interestingly, several currently available SSRIs have demonstrated safe and effective common doses in animal experiments. Multiple studies (Tables 2 and 4) have assessed the safety and efficacy of different injection methods and doses of SSRIs for AD treatment in animal models. For instance, fluoxetine administered orally at 5 mg/kg and via intraperitoneal injection at 10 mg/kg; citalopram administered via intraperitoneal injection at 10 mg/kg; and escitalopram administered orally at 10 mg/kg. This also suggests the potential of SSRIs in clinical research for AD, both as standalone therapeutic agents and as adjunctive therapies. It is crucial to note that the rate of drug absorption may vary significantly between intraperitoneal administration in mice and oral administration in humans. Consequently, direct comparison of doses may prove challenging. In general, SSRIs have shown promise in treating the pathologies and comorbidities associated with AD. This may represent a promising avenue for therapeutic intervention in patients with AD, particularly in the context of integrated management of the condition and improvements in quality of life.

Recently, an ongoing clinical trial has proposed a multimodal precision prevention strategy combining lifestyle modifications and metformin to prevent cognitive impairment and disability [209]. This strategy bridges the gap between pharmacological and non-pharmacological interventions for the prevention of dementia, providing a comprehensive prevention approach. Another ongoing clinical trial is investigating the effects of two lifestyle interventions on brain health. Both interventions emphasize exercise, dietary modifications, cognitive and social stimulation, and cardiovascular health [210]. Traditional single-target studies for treating AD often fail to achieve the desired outcomes. Therefore, current research increasingly focuses on multi-targets and multiple-intervention strategies to comprehensively address the complex pathological mechanisms of AD and its complications comprehensively. This comprehensive intervention strategy aligns better with the multifaceted pathological characteristics of AD and is expected to yield more significant results in both prevention and treatment. Given the significant role of 5-HT-related drugs in improving AD and its complications, these agents can also be integrated into this intervention strategy to simultaneously address multiple pathological mechanisms. For example, SSRIs have demonstrated positive effects in the treating vascular cognitive impairment, AD, and various complications associated with AD [38, 140, 211, 212].

## 5. Reflections based on exercise integration biology: potential mechanisms of the 5-HT system in exercise to improve AD

It is well known that AD is not caused by a single factor; its pathogenesis involves a complex, multifaceted interaction among genetic, environmental, lifestyle, and neurobiological factors. This complexity also contributes to the common comorbidities associated with AD, such as depression and type 2 diabetes. Exercise has received significant attention as a non-pharmacological intervention that can provide systemic and multifaceted physical health benefits. The integrative biology of exercise posits that physical activity challenges the body's internal environment, disrupting homeostasis and prompting response at the cellular, tissue, and organ levels. This integration and response at multiple levels establishes a new dynamic equilibrium for the organism, enhancing muscle energy and oxygen supply to meet the demands of muscle contraction. This disruption and remodeling of systemic homeostasis not only benefit skeletal muscle but also positively affect multiple organs and systems in the body [18]. 5-HT is a neurotransmitter that is widely distributed both peripherally and centrally and is significantly influenced by exercise. Exercise can influence the 5-HT system in various cells, tissues, and organs, enabling these systems to fulfill distinct physiological roles. BDNF and inflammatory mediators can cross the BBB through the peripheral circulation and exert effects on brain tissue. Based on the integrative biology of exercise and the multifaceted, systemic nature of 5-HT, this section further discusses the potential mechanisms by which exercise may ameliorate AD through 5-HT-mediated peripheral-central crosstalk.

### 5.1 Exercise improves crosstalk mechanisms in AD: 5-HT and energy metabolism

#### 5.1.1 5-HT and glucose metabolism

Disorders of glucose metabolism frequently coincide with the development of AD pathology and can exacerbate its progression [213]. Moreover, individuals with type 2 diabetes have an increased risk of developing AD [214]. Therefore, improving glucose metabolism disorders could potentially yield beneficial effects for both preventing and treating AD. Research indicates that disruptions in brain glucose metabolism are linked to impairments in the PI3K/Akt signaling pathway, which is downstream of insulin. These disruptions are associated with reduced levels of insulin, its receptor, and insulin-like growth factor (IGF-1) [215]. Notably, exercise may regulate insulin and IGF-1 levels by influencing the 5-HT system, which subsequently regulates glucose metabolism. Firstly, within pancreatic islets, 5-HT may enhance insulin secretion through the serotonylation of GTPases in pancreatic β-cells [216-218]. Since central insulin primarily derives from peripheral circulation [219], the peripheral 5-HT system plays a crucial role in insulin secretion. This has important implications for brain glucose metabolism, which is influenced by exercise-mediated modulation of the 5-HT system. Secondly, abnormalities in the PI3K/Akt/mTOR signaling pathway in the AD brain also impair glucose transporter proteins (GLUTs), hindering normal glucose transport. This reduction in glucose uptake and transport, combined with decreased activity of key enzymes for oxidative catabolism, contributes to impaired glucose metabolism [220]. Moreover, disruptions in this pathway suppress O-N-acetylglucosamine (O-GlcNAc) glycosylation, activating GSK-3α/β, which further enhances Aβ production and Tau protein phosphorylation [221]. Eight weeks of treadmill exercise have been shown to increase the synthesis of Trp hydroxylase in the brains of mice on a high-fat diet, thereby promoting the production of 5-HT and subsequently modulating the activity of the PI3K/Akt signaling pathway [222]. IGF-1 is hypothesized to participate in the process through which exercise modulates the PI3K/Akt signaling pathway via 5-HT. Specifically, 5-HT can activate CREB/Akt signaling through 5-HT_7_R in hepatocytes, thereby influencing the synthesis and secretion of IGF-1 [223]. IGF-1 has been shown to cross the BBB to promote cell proliferation and BDNF gene expression in the dentate gyrus of the hippocampus [224]. Additionally, it plays a crucial role in the brain's key pathway of insulin regulation of glucose uptake, the PI3K/Akt/mTOR pathway [215]. In summary, the 5-HT system likely influences glucose metabolism through its regulation of insulin and IGF-1 levels. Exercise potentially enhance these effects and may exert central positive effects through mechanisms involving islet-brain and liver-brain crosstalk.

#### 5.1.2 5-HT and lipid metabolism

In addition to glucose metabolism, extensive clinical investigations and epidemiological studies have established a close relationship between lipid metabolism disorders and the pathogenesis and progression of AD [225-227]. Diseases associated with obesity, such as hypercholesterolemia, atherosclerosis, and non-alcoholic fatty liver disease, are recognized as risk factors for AD [228-231]. These diseases are often associated with peripheral lipid metabolism disorders, which can lead to peripheral inflammation and insulin resistance. Such disturbances can eventually affect central inflammation and insulin resistance [232]. Additionally, inflammatory factors can affect the permeability of the BBB, allowing increased cholesterol from the peripheral to cross through the BBB, which in turn influences central lipid metabolic homeostasis. Thus, modulation of peripheral lipid metabolism may have a positive effect on the treatment of AD.

Exercise, as a crucial physiological stimulus, promotes the synthesis and turnover of 5-HT through its involvement in lipid metabolic processes. This enhancement further amplifies the beneficial effects of 5-HT in AD therapy. Moreover, 5-HT plays a role in regulating lipid metabolism, establishing a beneficial cycle that mitigates the pathological progression of AD. Specifically, during exercise, lipolysis alters the distribution of Trp in plasma through adrenergic-induced release of free fatty acids [233]. This physiological process induces the release of albumin, which subsequently increases the level of free Trp in the blood, prompting freer Trp to enter the brain. This enhances both the central and peripheral 5-HT systems, effectively alleviating the limited ability of peripheral 5-HT to cross the BBB, thereby promoting the beneficial role of 5-HT in improving AD. Additionally, peripheral triglycerides, free fatty acids and certain forms of cholesterol can influence central lipid metabolic homeostasis through the BBB [234]. Specifically, triglycerides can impair cognition and modulate microglial activity in the brain [235, 236]. Free fatty acids, particularly those that are peroxidized, have detrimental effects on cellular function [237]. Excessive accumulation of cholesterol in the brain, along with a high-cholesterol diet, is associated with Aβ deposition and Tau hyperphosphorylation [238-241]. Exercise increases peripheral 5-HT levels, which in turn elevates circulating concentrations of bile acids, reduces plasma triglycerides, cholesterol, and unesterified fatty acid concentrations, thereby promoting lipid metabolism. Ultimately, this alleviates the aforementioned processes [242]. These studies suggest that 5-HT can interact with lipid metabolic processes to improve both peripheral and central lipid metabolic homeostasis and exert cerebroprotective functions. Furthermore, exercise can enhance this potential role.

### 5.2 Exercise improves crosstalk mechanisms in AD: 5-HT and gut homeostasis

The maintenance of gut homeostasis, characterized by the balance of gut microbiota, is closely linked to AD. The relationship is currently under active investigation within the scientific community. Disruption of the microbiota, leading to increased permeability of the gut and BBB, may contribute to the development and progression of AD and other age-related neurodegenerative diseases [243]. Evidence from Mendelian randomization (MR) studies support a potential causal relationship between the gut microbiome and AD [244]. Approximately 95% of the body's 5-HT is found in the gut. Moreover, MR studies have demonstrated that 5-HT exerts a protective effect against neurodegenerative diseases such as Parkinson's disease [245]. Therefore, maintaining gut homeostasis is crucial for the therapeutic approach to AD, with 5-HT emerging as a potential therapeutic factor. Dysregulation of the gut microbiota may increase permeability, thus impacting CNS health. Given the widespread presence of 5-HT in the gut and its protective role in neurodegenerative diseases, it represents a promising therapeutic target for modulating gut homeostasis.

The gut-brain axis is a bidirectional communication network between the gastrointestinal tract and the CNS. Many studies have been conducted to explore the important role of the gut-brain axis in neurodegenerative disorders. 5-HT plays a crucial role in stimulating the enteric nervous system and modulating gastrointestinal function. In particular, 5-HT exerts a protective effect on the gut through stimulation of 5-HT_4_R, positively influencing the development and survival of enteric neurons. 5-HT_4_R knockout mice exhibit a loss of enteric neurons within the first few months of life, suggesting that 5-HT_4_R-mediated 5-HT signaling is essential for maintaining the normal state of enteric neurons. Additionally, many other 5-HTR subtypes and selective agents play a role in regulating of gastrointestinal motility, secretion, and sensation [246]. These further underscores the importance of the 5-HT system in intestinal homeostasis.

**Figure 2.**
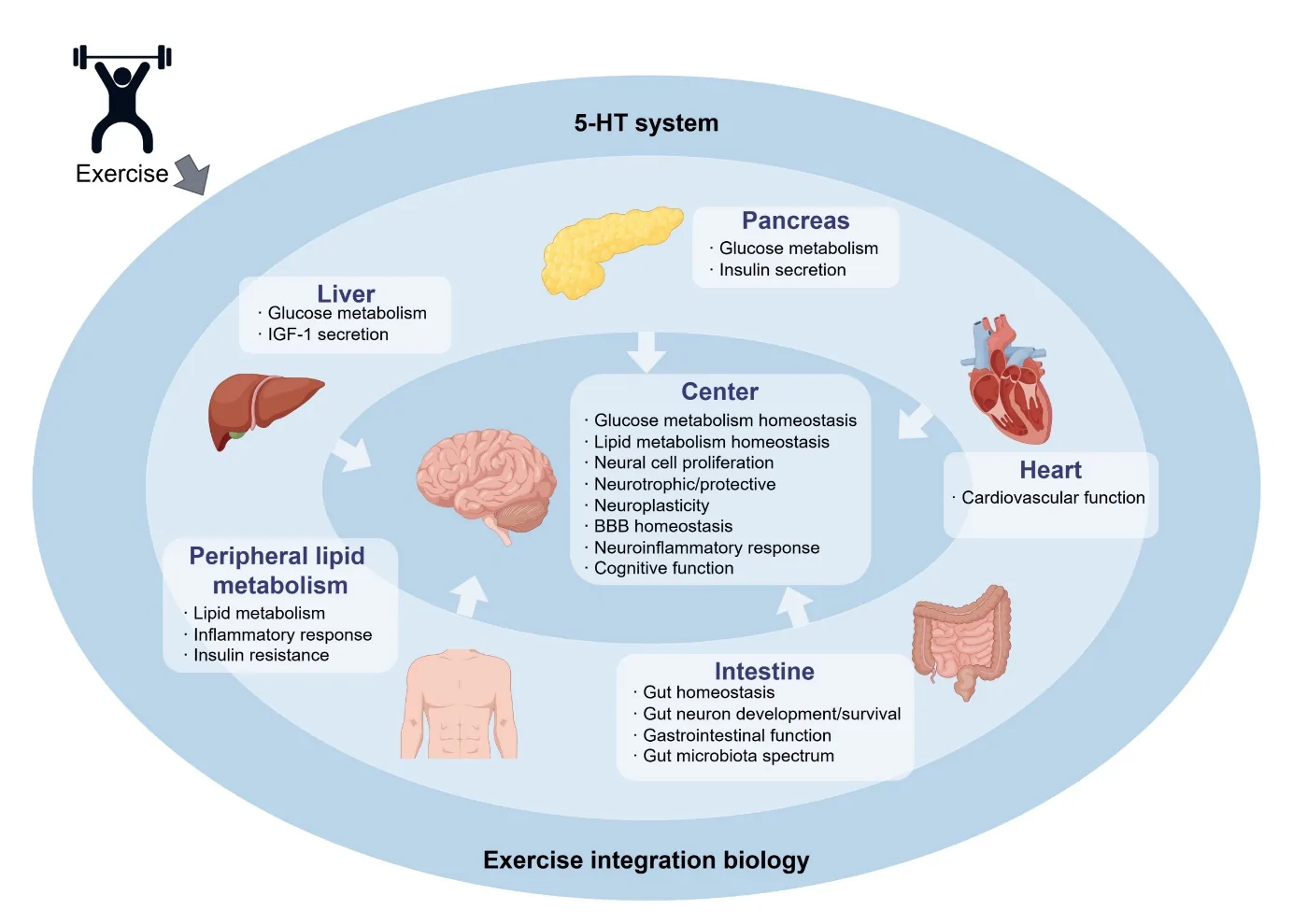
**Schematic representation of mechanisms of peripheral-central crosstalk mediated by the 5-HT system in the perspective of integrative biology of exercise**. During exercise, the 5-HT system can play a role in the regulation of the central system by the peripheral system through islet-brain, liver-brain, peripheral lipid metabolism-brain, gut-brain and heart-brain dialogue mechanisms (as shown in the figure).

Exercise enhances the role of 5-HT in gut function by increasing peripheral 5-HT levels [247]. Exercise training modifies the gut microbiota profile [248], which in turn influences the levels of gut peptides, including 5-HT. This modulation affects vagal afferent pathways, thereby influencing gut metabolism through the microbiota-gut-brain axis [249]. Exercise-induced changes in gut flora exert beneficial effects on cognition and mood through 5-HT signaling [250]. This is possibly because 5-HT and its precursor 5-hydroxytryptophan (5-HTP), produced by gut bacteria, transmit signals to the brain via receptors on intestinal mucosal cells and vagus nerves [251], exerting a crosstalk with the brain via the gut-brain axis. In summary, 5-HT can interact with gut flora to exert positive effects such as intestinal homeostasis, gut protection and improved cognition, a process facilitated by exercise.

Additionally, gut homeostasis plays a crucial role in cardiovascular health [252]. While current research has not specifically examined the impact of exercise-mediated 5-HT on cardiovascular function, it is known to affect cardiovascular function [253]. Several studies have demonstrated that improvements in cardiovascular health may positively influence AD and cognitive function [254]. Therefore, it is suggested that exercise may ameliorate brain dysfunction through 5-HT-mediated mechanisms, facilitating heart-brain crosstalk.

It is noteworthy that the aforementioned components are not independent of AD but are interconnected. For instance, glucose and fatty acids can stimulate the release of 5-HT, which enhances intestinal motility and nutrient absorption [217]. Additionally, exercise training can mitigate obesity-induced inflammatory pathways by modifying the gut microbiota [248]. AD, the most prevalent neurodegenerative disorder globally, involves complex pathogenetic mechanisms and complications. Conventional single-target therapies have demonstrated limited efficacy. The positive role of exercise as a non-pharmacological treatment in managing AD may stem from the systemic health benefits of exercise. 5-HT is widely present as a neurotransmitter both peripherally and centrally. During exercise-regulated 5-HT system, 5-HT can exert AD-improving and pro-cognitive effects by acting on peripheral tissues or physiological processes through peripheral-central crosstalk mechanisms (Fig 2).

## 6. Conclusions and future perspectives

The 5-HT system is widespread in the central and peripheral nervous systems and plays a systemic regulatory role in the amelioration of AD. It improves the pathological processes of AD by regulating Aβ production and clearance, modulating Tau phosphorylation, alleviating neuroinflammation, and increasing the levels of both central and peripheral ACh and BDNF. Additionally, it enhances the interaction between 5-HT and these two neurotransmitters.

Based on the numerous similarities between the effects of exercise on the 5-HT system and those of SSRIs, we found that the 5-HT system mediates the beneficial effects of exercise through various potential mechanisms for AD. We elaborated on the important role of the 5-HT system in the prevention and treatment of AD. Given the integrative biology of exercise, the 5-HT system mediates the regulation of glucose metabolism, lipid metabolism, and intestinal homeostasis during exercise. Furthermore, it may ameliorate brain environmental disorders through peripheral-central crosstalk mechanisms, such as pancreas-brain, liver-brain, gut-brain, and heart-brain interactions. These findings provide new perspectives and approaches for the prevention and treatment of AD.

Although numerous studies have elucidated the mechanisms by which exercise improves 5-HT levels in AD, several unresolved issues remain.

1. Exercise training strongly stimulates 5-HT levels, and the influence of exercise on regulation of the 5-HT system shows specificity across different brain regions and tissues, being affected by exercise patterns and intensities. This specificity can influence various physiological functions, thereby impacting AD. Future research should focus on the correlational effects of different intensities, durations, and types of exercise on 5-HT system and the mechanisms related to the regulation of AD. These studies should be conducted in both animal models and clinical settings. Additionally, various 5-HT-related drugs have also demonstrated positive effects in the treatment of AD. Future studies may also explore the effects of exercise combined with related pharmacological agents (e.g., SSRIs) in AD. For instance, a RCT with a three-arm design could investigate the effects of AD medications on exercise training compared to a control group or examine the combination of exercise and pharmacological treatment to determine whether the intervention effects are synergistic or antagonistic, and whether these combined effects persist.
2. The regulatory role of 5-HT in the cardiovascular system is well-established, and exercise may also play a role. Therefore, whether the regulatory effect of exercise on cognitive function is partially achieved by its influence on vascular function warrants further exploration.
3. Although the development of drugs targeting the 5-HT system for cognitive impairment holds great promise, some clinical trials of 5-HT-related drugs have not yielded satisfactory results, which may be attributed to factors such as the experimental subjects, sample size, drug dosage and duration of the clinical trials. A systematic review of clinical studies of SSRIs in AD indicates that the lack of evidence supporting SSRIs as cognitive enhancers or disease modulators in AD results from deficiencies in clinical trial design rather than the reporting of negative results [255]. The results of drug research are influenced by multiple factors, including experimental design, animal models, administration timing and methods, as well as dosage. This complexity emphasizes the importance of considering the variability of these factors when interpreting research results. Future studies should give priority to these factors to ensure more accurate and reproducible experimental results. Several issues still need to be addressed before the clinical translation of SSRIs, including the need for more RCTs on patients with or at risk of AD, as well as longer follow-up periods to clarify the dose-effect relationship and potential side effects of SSRIs in AD prevention. Additionally, more clinical trials are needed in the future for longitudinal studies to fully understand the safety and efficacy of long-term SSRI treatment. Furthermore, further exploration of the accuracy of dose conversion of SSRIs between different species, particularly considering the differences in drug absorption across various administration methods, will greatly assist preclinical and clinical trials. Researchers should also focus on the efficacy differences of SSRIs across different genetic backgrounds and pathological stages, to provide more accurate dosing guidelines for individualized treatment.

In conclusion, an in-depth exploration and resolution of these issues will enable a deeper understanding of the integrated biological principles of exercise for brain health.
